# Transgene expression in mice of the Opa1 mitochondrial transmembrane protein through bicontinuous cubic lipoplexes containing gemini imidazolium surfactants

**DOI:** 10.1186/s12951-021-01167-x

**Published:** 2021-12-18

**Authors:** Mónica Muñoz-Úbeda, Martina Semenzato, Anais Franco-Romero, Elena Junquera, Emilio Aicart, Luca Scorrano, Iván López-Montero

**Affiliations:** 1 Instituto de Investigación Biomédica Hospital, 12 de Octubre (imas12), Madrid, Spain; 2 Dpto. Química Física, Universidad Complutense de Madrid, Madrid, Spain; 3 Fondazione Per La Ricerca Biomèdica Avanzata, Venetian Institute of Molecular Medicine (VIMM), Padova, Italy

**Keywords:** Gemini cationic lipids, Gene therapy, Opa1 mitochondrial protein, CD-1 mouse model

## Abstract

**Background:**

Lipoplexes are non-viral vectors based on cationic lipids used to deliver DNA into cells, also known as lipofection. The positively charge of the hydrophilic head-group provides the cationic lipids the ability to condensate the negatively charged DNA into structured complexes. The polar head can carry a large variety of chemical groups including amines as well as guanidino or imidazole groups. In particular, gemini cationic lipids consist of two positive polar heads linked by a spacer with different length. As for the hydrophobic aliphatic chains, they can be unsaturated or saturated and are connected to the polar head-groups. Many other chemical components can be included in the formulation of lipoplexes to improve their transfection efficiency, which often relies on their structural features. Varying these components can drastically change the arrangement of DNA molecules within the lamellar, hexagonal or cubic phases that are provided by the lipid matrix. Lipofection is widely used to deliver genetic material in cell culture experiments but the simpler formulations exhibit major drawbacks related to low transfection, low specificity, low circulation half-life and toxicity when scaled up to in vivo experiments.

**Results:**

So far, we have explored in cell cultures the transfection ability of lipoplexes based on gemini cationic lipids that consist of two C_16_ alkyl chains and two imidazolium polar head-groups linked with a polyoxyethylene spacer, (C_16_Im)_2_(C_4_O). Here, PEGylated lipids have been introduced to the lipoplex formulation and the transgene expression of the Opa1 mitochondrial transmembrane protein in mice was assessed. The addition of PEG on the surface of the lipid mixed resulted in the formation of Ia3d bicontinuous cubic phases as determined by small angle X-ray scattering. After a single intramuscular administration, the cubic lipoplexes were accumulated in tissues with tight endothelial barriers such as brain, heart, and lungs for at least 48 h. The transgene expression of Opa1 in those organs was identified by western blotting or RNA expression analysis through quantitative polymerase chain reaction.

**Conclusions:**

The expression reported here is sufficient in magnitude, duration and toxicity to consolidate the bicontinuous cubic structures formed by (C_16_Im)_2_(C_4_O)-based lipoplexes as valuable therapeutic agents in the field of gene delivery.

**Graphical Abstract:**

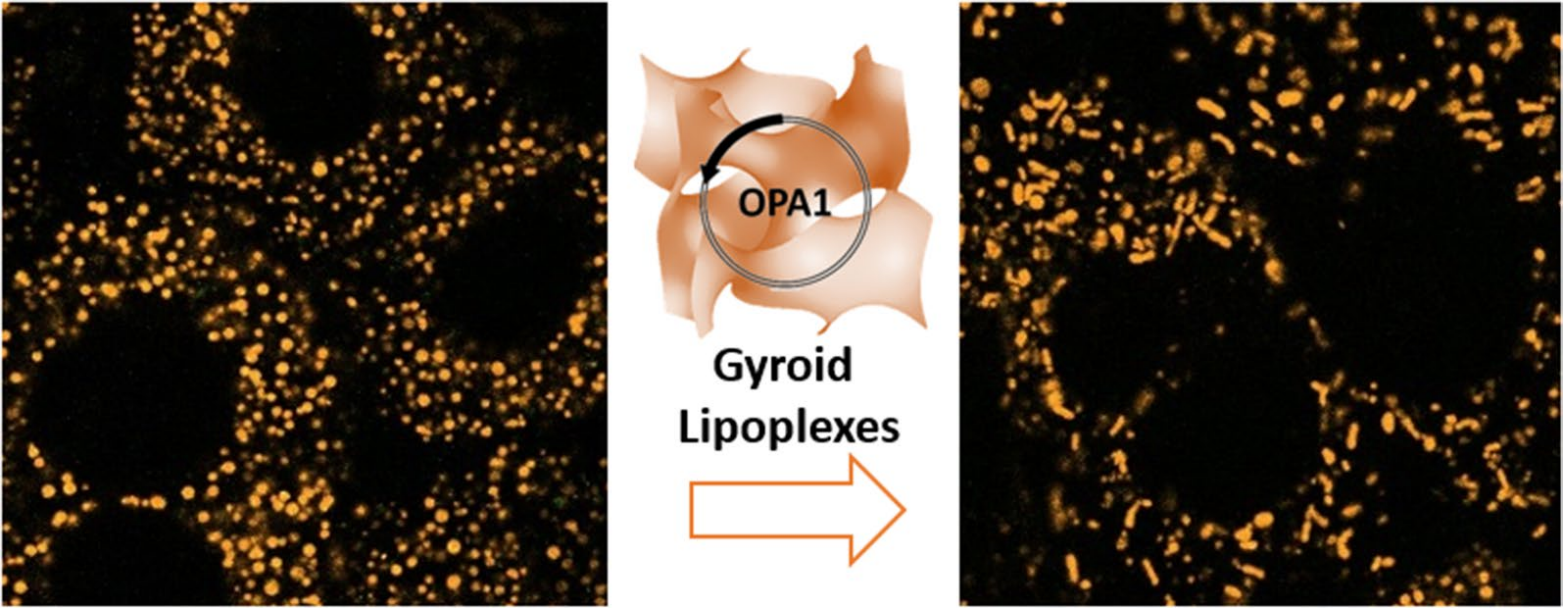

**Supplementary Information:**

The online version contains supplementary material available at 10.1186/s12951-021-01167-x.

## Background

Since the gene therapy emerged in the early 60s, the main objective of treating a wide variety of genetic disorders has been addressed either by the introduction of exogenous DNA to supply damaged cellular DNA or by inserting functional genes in living cells [1]. However, gene delivery follows a complex pathway where each step must fulfill specific constrains. Briefly, DNA binding and loading to biocompatible and stable nanovehicles is crucial for its cell uptake, endosomal escape and nuclear delivery for transcription. DNA is a negatively charged and rigid molecule that specifically interacts with positive nanocarriers through electrostatic interactions and allows the condensation of DNA molecules to cross the negatively charged plasma membrane. Among the most widely used cationic and non-viral vectors; polymers, dendrimers and lipids offer a flexible manufacturing, versatile tailoring and safety profiles [2, 3].

Lipoplexes, which consist of a lipid/DNA highly packed complex, are obtained by self-assembling in aqueous solution [4], giving rise to different phases [5, 6]. Lipid-based phases allow not only the specific incorporation of multiple drugs along with DNA into its hydrophilic or hydrophobic cores [7] but also versatile formulations able to simultaneously address cell targeting, highly concentrated loads and an enhanced endosomal escape [8]. In particular, inverted hexagonal phases display better transfection efficiency than lamellar phases [6]. Inverted hexagonal phases result of the presence of non-bilayer lipids such as 1,2-dioleoyl-sn-glycero-3-phosphoethanolamine (DOPE) [9, 10], which also facilitates protein-independent membrane destabilization and fusion and subsequent endosomal escape [9, 10]. Other mesomorphic structures can be obtained by means of other helper lipids such as monoolein glycerol (MOG). MOG is the most commonly used cubic phase forming lipid [11]. Cubic phases have proved to be promising in gene transfer, especially in the field of siRNA silencing that is favored by membrane fusion mediated by the onset of pore formation [12, 13]. Although lipoplexes have been shown as efficient DNA carriers in living cells [7, 14] clinical studies using lipoplexes are still scarce. It is believed that lipoplexes, similarly to liposomes, can be rapidly recognized and removed from the circulation by the reticuloendothelial system (RES) [15] when administrated in animal models, thus sinking any significant localization in targeted cells [16]. Since three decades ago, it is known that polyethyleneglycol (PEG)-coated liposomes improve their stability and increase their half-lives in circulation [17]. PEG coating inhibits protein adsorption and opsonization of liposomes, thereby avoiding or retarding liposome recognition by the RES. PEGylation has been previously used to produce new long-circulating lipoplexes for gene delivery purposes [18–20].

So far, we have explored the use of synthetic gemini cationic lipids with two positive polar heads linked by a spacer from different length ((C_16_Im)_2_(C_4_O)), and mixed with the zwitterionic phospholipid DOPE as efficient DNA carriers [21–25]. Lipids with multiple cationic head groups display higher transfection efficiency in comparison with conventional cationic lipids bearing only one polar head. [14, 24] Lipoplexes containing gemini cationic surfactants were shown to complex, transport and deliver into the nucleus circular plasmids coding not only for cytosolic proteins such as GFP or luciferase [23–25], but also the outer mitochondrial membrane fusion protein Mfn1 [26]. Here, we incorporate in the lipoplex formulation the current gold standard for nanoparticle furtiveness coating by means of commercially available PEGylated lipids (DSPE-PEG). The (C_16_Im)_2_(C_4_O)/DOPE/DSPE-PEG mixed lipids condensed a DNA plasmid coding for the mitochondrial fusion protein Opa1 into a Ia3d bicontinuous cubic phase as determined by small angle X-ray scattering (SAXS).

Mitochondria are dynamic interconnected organelles that undergo fusion and fission processes that regulate the energetic levels of the cell [27]. In mammals, Opa1 is the transmembrane GTPase protein involved in the Inner Mitochondrial Membrane (IMM) fusion [28]. Opa1 deletion produces mitochondrial fragmentation and causes the Autosomal Dominant Optic Atrophy (ADOA) mitochondrial disease [29]. The exogenous Opa1 expression can restore the mitochondrial fusion and rescue the mitochondrial phenotype from fragmented to elongated mitochondrial network [30]. This straightforward observable is a powerful tool for the characterization of mitochondrial morphology under normal and pathogenic conditions [31]. Prior to the in vivo experiments, we demonstrate the ability of (C_16_Im)_2_(C_4_O)/DOPE/DSPE-PEG/OPA1 lipoplexes to rescue the mitochondrial phenotype in OPA1-Knockout mouse embryonic fibroblasts (OPA1-KO MEFs). The phenotype recovery was accompanied with the overexpression of Opa1 protein. After intraperitoneal, intracardiac or intramuscular administration, we monitored the biodistribution of lipoplexes and the transgene expression of the mitochondrial fusion protein Opa1 in CD-1 mice. As expected and independently of the route of administration, the labeled lipoplexes remained mainly localized for 48 h in vital tissues traditionally ascribed for drug elimination or metabolism as liver, spleen or kidneys. However, the (C_16_Im)_2_(C_4_O)/DOPE/DSPE-PEG/OPA1 lipoplexes were able to penetrate in other tissues with tight endothelial barriers such as brain, heart, and lungs upon intramuscular administration. The Opa1 levels were also increased in those tissues as measured by either western blotting or by RNA expression analysis through RT-PCR.

Along with our previous works, the control over the cationic to fusogenic lipid ratio, the lipid to DNA ratio as quantified by the effective charge ratio, the supramolecular arrangement within the lipoplex and more important, the route of administration make this work a first experimental approach to a preclinical study for the validation of lipoplexes as valuable therapeutic agents in gene delivery against mitochondrial disorders [32].

## Results and discussion

### Formation of (C_16_Im)_2_(C_4_O)/DOPE/DSPE-PEG/OPA1 lipoplexes and structural characterization

The mixed lipids used in this work consisted of bis (hexadecyl dimethyl imidazolium) oxyethylene gemini cationic lipid (with two imidazolium polar head linked with a polyoxyethylene spacer of one oxygen and four carbon atoms, (C_16_Im)_2_(C_4_O) [24], the zwitterionic phospholipid DOPE and the PEGylated lipid (1,2-distearoyl-sn-glycero-3-phosphoethanolamine-N-[carboxy(polyethylene glycol)-2000]) (DSPE-PEG) in a 15:80:5 molar ratio. The cationic lipid is necessary to guarantee the condensation of the negatively charged plasmid through electrostatic interactions. The non-bilayer DOPE is known to form hexagonal phases [9, 10] that facilitates membrane destabilization and fusion and subsequent endosomal escape. DSPE-PEG was used to prevent the adsorption of serum proteins and antibodies onto the surface of lipoplexes and render them furtive within the blood stream in in vivo experiments [17]. The mixed nanoaggregates, after hydration and extrusion, presented a hydrodynamic diameter of 200 ± 20 nm as measured by Dynamic Light Scattering and a zeta-potential of ξ = 28.2 ± 0.6 mV.

**Fig. 1:**
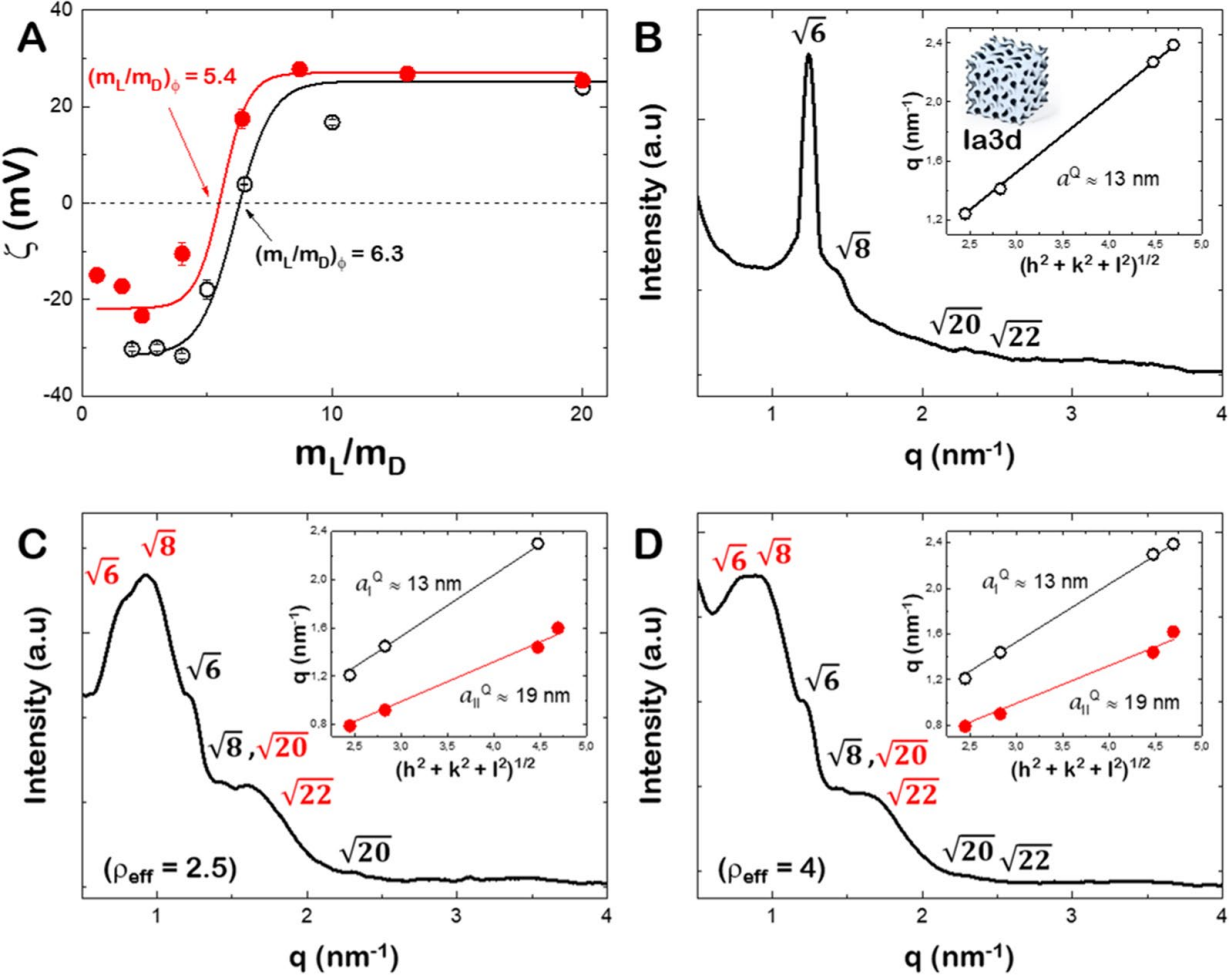
**A** Zeta potential of GCL/DOPE/DSPE-PEG/DNA at different Lipid/DNA mass ratios (mL/mD). (mL/mD)_Φ_, is the electroneutrality value. Black open circles for ctDNA and red circles for MSCV-OPA1. **B** SAXS diffractograms of GCL/DOPE/DSPE-PEG mixed lipids. Inset: q-values vs. ${\left({h}^{2}+{k}^{2}+{l}^{2}\right)}^{1/2}$ for the Ia3d phase. **C** SAXS diffractograms of GCL/DOPE/DSPE-PEG/OPA1 at ρ_eff_ = 2.5. Inset: q-values vs. ${\left({h}^{2}+{k}^{2}+{l}^{2}\right)}^{1/2}$ for two different Ia3d phases (red and white symbols). **D** SAXS diffractograms of GCL/DOPE/DSPE-PEG/OPA1 at ρ_eff_ = 4. Inset: q-values vs. ${\left({h}^{2}+{k}^{2}+{l}^{2}\right)}^{1/2}$ for two different Ia3d phases (red and white symbols)

Lipoplexes were formed by incubating the mixed lipids (C_16_Im)_2_(C_4_O)/DOPE/DSPE-PEG with increasing amounts of the plasmid DNA coding for the mitochondrial fusion protein Opa1 (MSCV-OPA1). The zeta potential curve of (C_16_Im)_2_(C_4_O)/DOPE/DSPE-PEG/OPA1 lipoplexes at different total lipid/DNA (m_L_/m_D_) mass ratio is plotted in Fig. 1A. At very high m_L_/m_D_ values, the zeta-potential value is close to the value measured for the mixed lipids alone. The zeta potential displays a charge inversion from positive to negative values as the m_D_ increases, and thus reaching a maximal negative value for DNA alone. The experimental points were fitted to a sigmoidal function and the zero potential value, (m_L_/m_D_)_Φ_, defines the electroneutrality, where the positive (C_16_Im)_2_(C_4_O) and the negative DNA charges are balanced (± effective charge ratio equals to 1, ρ_eff_ = 1). In general, increasing the cationic lipoplex charge (ρ_eff_ > 1) prevents particle aggregation that leads to a better interaction with the cell membrane and improves the transfection yield [24, 25, 33]. As in previous reports, the determination of the effective charge ratio between the positive charges given by the cationic lipids and the negative ones given by the phosphates of plasmid DNA, is crucial for preparing the different lipoplex formulations. To obtain the effective charge it is necessary to fit the zeta potential values for different mixed lipids/plasmid DNA mass ratio to a sigmoidal function in order to obtain the electroneutrality value when zeta potential is zero (more details in Additional file 1) [21]. The supercoiled conformation of plasmid DNA (pDNA) and the presence of a large amount of counterions usually render the DNA molecules with a lower effective negative charge than its nominal one. Also, the (C_16_Im)_2_(C_4_O) lipid used here have a delocalized positive charge that might yield also to a lower effective positive charge. In consequence, the effective charge of the cationic lipid (${q}_{{eff, L}^{+ }}^{+}$) and the plasmid DNA (${q}_{eff, D}^{-}$) are required for an accurate formulation of lipoplexes in terms of charge ratio. For that, the effective charge of (C_16_Im)_2_(C_4_O) is first determined in (C_16_Im)_2_(C_4_O)/DOPE/DSPE-PEG mixed lipids using a linear calf-thymus DNA (ctDNA) (see reference [21] for full details of the whole procedure). From the experimental value of (m_L_/m_D_)_Φ_ as measured from the zeta potential (Fig. 1A) and assuming ${q}_{linearD}^{-}$= − 2/bp, the obtained value for ${q}_{{eff, L}^{+ }}^{+}$ was + 1.9 ± 0.1 [24, 25]. With those values and the experimental value of (m_L_/m_D_)_Φ_ for (C_16_Im)_2_(C_4_O)/DOPE/DSPE-PEG/OPA1, we quantified the negative effective charge per base pair accessible to the (C_16_Im)_2_(C_4_O) lipid. We obtained ${q}_{eff, D}^{-}$ = − 1.8 ± 0.2/bp. This value is more negative than that found previously for other plasmid DNAs [21, 22, 24, 25] but similar to a plasmid DNA coding for an outer mitochondrial fusion protein Mfn1 [26]. In the following, we tested the transfection efficiency of lipoplexes prepared at ρ_eff_ = 2.5 and 4 where the positive charge of lipoplexes is ensured.

We have previously allocated, for a very similar system ((C_16_Im)_2_(C_4_O)/DOPE/MFN1 at ρ_eff_ = 4), a better transfection efficiency for lipoplexes structured into two compacted hexagonal H_II_ phases structure [26]. The addition of a PEGylated lipid into the mixed lipids might modify the supramolecular structure of lipoplexes, and thus modify the transfection efficacy. The aggregation pattern of the mixture of lipids, as well as the structure of the lipoplexes at ρ_eff_ = 2.5 and 4 were determined by SAXS experiments [5]. Figure 1B shows the radially integrated SAXS diffractogram for the ternary lipid mixture (C_16_Im)_2_(C_4_O)/DOPE/DSPE-PEG. We used the ratios of q-values corresponding to each diffraction peak to determine the identity of the phases present in the system [34]. The Bragg peaks found in the diffractograms for the mixed lipids in the absence of OPA1 follow the ratios of $\sqrt{6}$, $\sqrt{8}$,$\sqrt{14}$, $\sqrt{16}$,$\sqrt{20}$, $\sqrt{22}$, etc.; which are indexed to a bicontinuous cubic phase of the gyroid type (${Q}_{II}^{G}$, Ia3d). Due to the low signal to noise ratio of our samples, only the most intense peaks were resolved ($\sqrt{6}$, $\sqrt{8}$, $\sqrt{20}$ and $\sqrt{22}$). The large volume of the head group bearing the PEG polymer reduces the negative spontaneous curvature of the (C_16_Im)_2_(C_4_O)/DOPE/DSPE-PEG mixed lipids and might stabilize the phase transition from the hexagonal H_II_ phase found from the binary mixture [27] to a Ia3d cubic phase [11, 35]. The lattice periodic distance, ${a}^{Q}$, was determined through the linear relationship between the q-values and their corresponding ratio value as ${\left({h}^{2}+{k}^{2}+{l}^{2}\right)}^{1/2}$, where h, k and l are the Miller indices (see inset of Fig. 1B). The slope of the linear trend is $=\frac{2\pi }{{a}^{Q}}$ and a ${a}^{Q}$ value of 13.0 ± 0.1 nm was obtained. The periodic distance ${a}^{Q}$ comprises the hydrophobic region of lipids, $L$, and the radius of the water cylindrical channels where the pDNA might be hosted, ${r}_{w}$. Taking geometrical considerations [36] the ${r}_{w}$ can be obtained from ${r}_{w}=0.248{a}^{Q}-L$. If a value of $L$ ~ 1.7 nm is assumed for Ia3d cubic phases [35], the aqueous radius is ${r}_{w}$ ~ 1.5 nm.

The SAXS diffractograms of the (C_16_Im)_2_(C_4_O)/DOPE/DSPE-PEG/OPA1 lipoplexes show the presence of two coexisting cubic phases at both ρ_eff_ = 2.5 (Fig. 1C) and ρ_eff_ = 4 (Fig. 1D). A first gyroid phase is characterized by the same q-values and sharp peaks produced by the lipid scaffold (see Fig. 1B). The second Ia3d cubic phase displays broadened peaks that are also shifted to lower q-values. A ${a}^{Q}$ value of 19.0 ± 0.2 nm was obtained for this phase, which leads to swelled aqueous channels with radius ${r}_{w}$ ~ 2.8 nm. Surprisingly, the addition of MSCV-OPA1 did not compact the cubic phase through the electrostatic interactions between (C_16_Im)_2_(C_4_O)/DOPE/DSPE-PEG and the DNA, and the swollen cubic phase might result from steric hindrance after DNA hosting in the water channels. The positive counter ions accompanying the negatively charged DNA might induce electrostatic repulsion across the positively charged (C_16_Im)_2_(C_4_O)/DOPE/DSPE-PEG aggregates as previously reported [37]. As a result, some water channels of the gyroid cubic phase formed by the ternary mixture of (C_16_Im)_2_(C_4_O)/DOPE/DSPE-PEG are swollen by the presence of DNA plasmid [38].

### (C_16_Im)_2_(C_4_O)/DOPE/DSPE-PEG/OPA1 lipoplexes enter the cell through the endosomal pathway and present low cytotoxicity

**Fig. 2:**
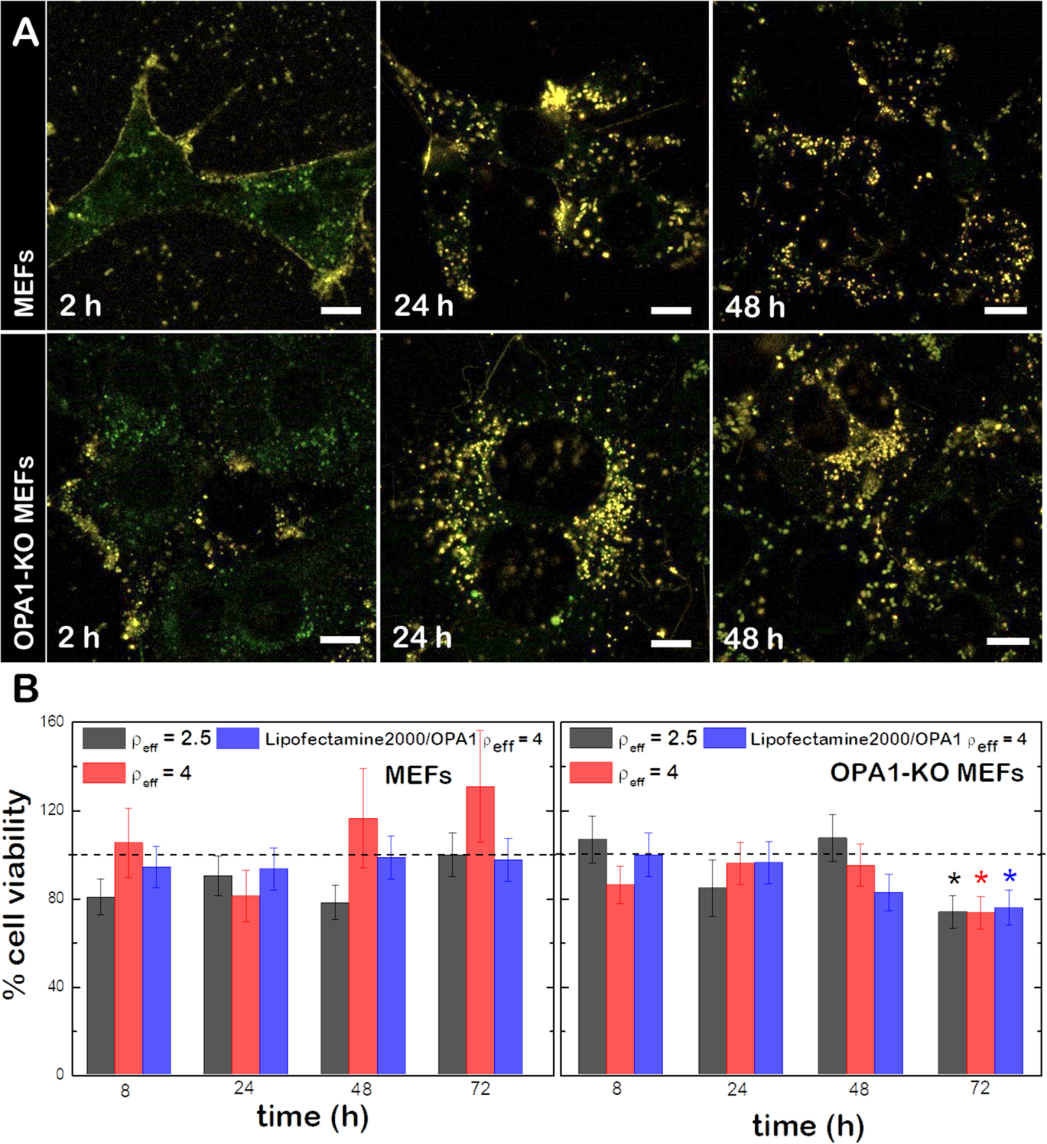
**A** Uptake of (C_16_Im)_2_(C_4_O)/DOPE/DSPE-PEG/OPA1 lipoplexes labeled with the fluorescent dye DiR’ (red channel) into MEFs wt and MEFs OPA1-KO MEFs (labeled with LysoTracker (green channel) at different incubation times. Yellow colors mean colocalization between channels. Scale bars are 10 μm. **B** Cell viability of MEFs wt (left) and OPA1-KO MEFs (right) upon incubation with (C_16_Im)_2_(C_4_O)/DOPE/DSPE-PEG/OPA1 lipoplexes at ρ_eff_ = 2.5 and 4 and Lipofectamine2000/OPA1 at ρ_eff_ = 4 as a positive control. Student’s t-test was performed to measure the significance of statistical difference between the different groups and the negative control (in the absence of treatment, dashed line). (*p < 0.05 was considered statistically significant)

The cellular uptake of lipoplexes and their cytotoxicity were evaluated in both wild type mouse embryonic fibroblasts (MEFs wt) and in OPA1-Knockout mouse embryonic fibroblasts (OPA1-KO MEFs). Prior to lipoplex formation, the ternary lipid mixture was complemented with 1% mol of the fluorescent dye DiR (red channel) and cells were labeled with a lysosomal marker (green channel, see “Methods”) to track the lipoplexes uptake within cells. Then, both MEFs wt and OPA1-KO MEFs were incubated with 130 µM of lipoplexes and imaged up to 24 h (Fig. 2A). During early uptake (2 h incubation) lipoplexes are only found at the proximity of plasma membranes as no colocalization of the lysosomal dye and the lipid probe is displayed. At longer incubation times (8 and 24 h), a progressive colocalization of the probes is observed. The maximum overlap of the green and the red fluorescence signals is monitored at 24 h, indicative for a complete colocalization of lipoplexes and lysosomes. We conclude that the lipoplex uptake was produced via the endosomal pathway. Complementary information was obtained through living cell imaging (Additional file 1: Fig. S1) where the cellular uptake in MEFs wt of lipoplexes at different concentration is revealed after 24 h incubation. Here, the incubation of cells with high concentrations of lipoplexes (400 µM) did not compromise cell viability or morphology.

To quantitatively determine the toxicity of (C_16_Im)_2_(C_4_O)/DOPE/DSPE-PEG/OPA1 lipoplexes, we exposed MEFs wt and OPA1-KO MEFs to lipoplexes at different ρ_eff_ and measured the cell viability over time by means of the resazurin-based alamarBlue cell viability assay (Fig. 2B). Lipofectamine2000 was used as a positive control. In general, (C_16_Im)_2_(C_4_O)/DOPE/DSPE-PEG/OPA1 lipoplexes either at ρ_eff_ = 2.5 or 4 do not produce a significant effect on cell viability after 48 h incubation. Only lipoplexes at ρ_eff_ = 4 presented a significant cytotoxicity (~ 20%, *p* < 0.05) on OPA1-KO MEFs after 72 h of incubation. The low cytotoxicity of (C_16_Im)_2_(C_4_O)/DOPE/DSPE-PEG/OPA1 lipoplexes after 24 h incubation was also confirmed independently by scanning flow cytometry (Additional file 1: Fig. S2). In comparison with other monomeric or dimeric cationic lipids, the low cytotoxicity of (C_16_Im)_2_(C_4_O)/DOPE/DSPE-PEG/OPA1 lipoplexes lays on their delocalized positive charge [24].

### The overexpression of Opa1 by the cubic (C_16_Im)_2_(C_4_O)/DOPE/DSPE-PEG/OPA1 lipoplexes partially rescues the elongated mitochondrial phenotype in OPA1-KO MEFs

**Fig. 3:**
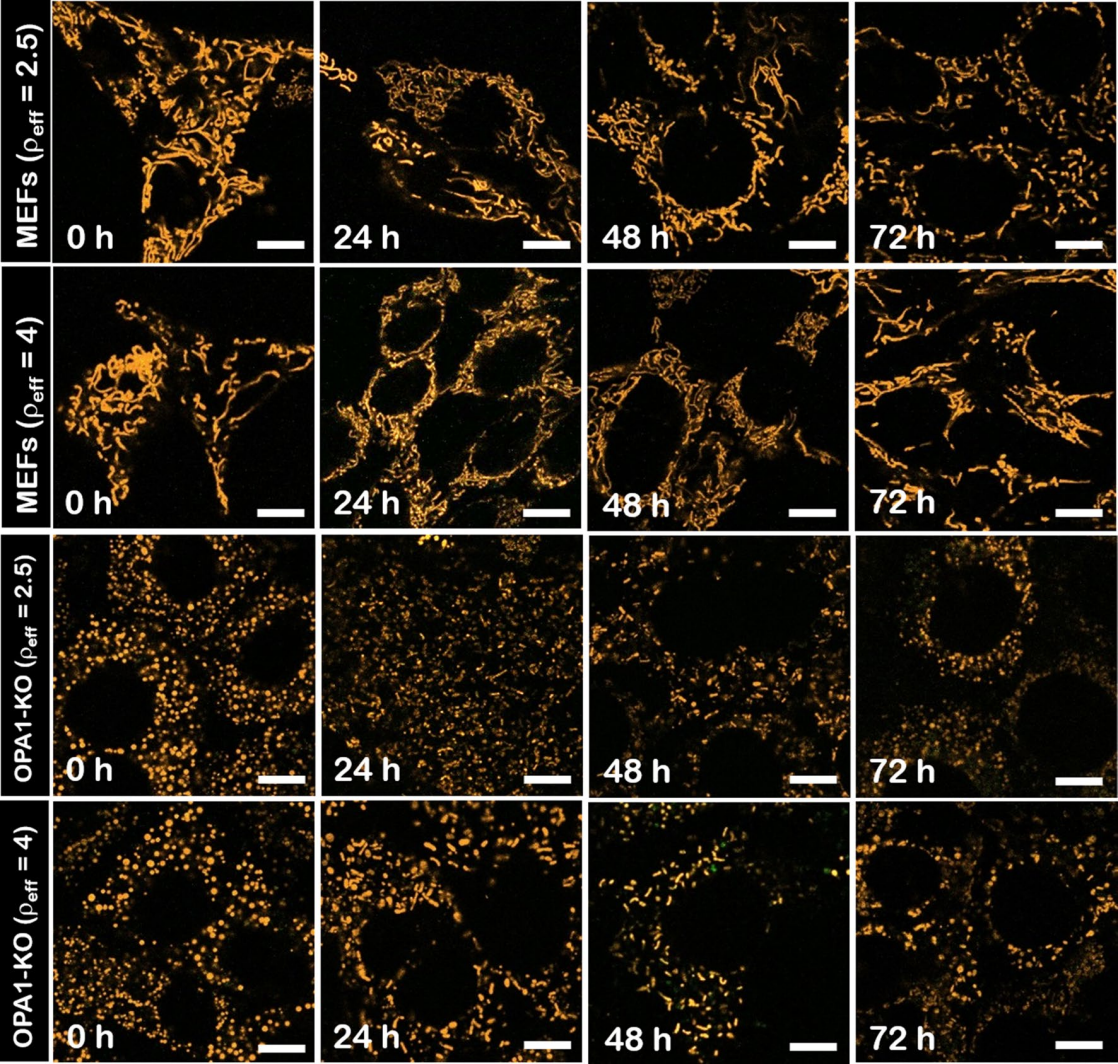
Confocal fluorescence microscopy images MEFs wt (upper rows) and OPA1-KO MEFs (lower rows) transfected with (C_16_Im)_2_(C_4_O)/DOPE/DSPE-PEG/OPA1 lipoplexes at ρ_eff_ = 2.5 and 4 at different time intervals. The mitochondrial network was labeled with TMRM (1 µM). Scale bars are 10 µm

In the absence of treatment, the morphology of the mitochondrial network is elongated or ball-shaped under normal (MEFs wt) and pathogenic conditions (OPA1-KO MEFs) respectively [32] (Fig. 3). Under the assumption that an exogenous expression of Opa1 can restore the mitochondrial fusion and complement the mitochondrial phenotype from fragmented to elongated mitochondrial network [31], the efficiency of (C_16_Im)_2_(C_4_O)/DOPE/DSPE-PEG/OPA1 lipoplexes was tested in both MEFs wt and OPA1-KO MEFs by scanning confocal fluorescence microscopy. As shown in confocal micrographs, MEFs wt maintained the normal mitochondrial phenotype 72 h after treatment with lipoplexes at ρ_eff_ = 2.5 and ρ_eff_ = 4. Though this is not a direct evidence for protein expression, this observation is in agreement with the non-toxicity of these lipoplexes (Fig. 3, top panel). As for OPA1-KO MEFs, the fragmented mitochondria were partially and progressively turned into elongated mitochondria from 8 h of treatment with lipoplexes.

For a better quantification of the mitochondrial phenotype complementation, the mitochondrial network structure was outlined from its underlying tree structure as determined by the Skeleton Analyzer plug-in from ImageJ. [26, 39] The ratio of elongated mitochondria over the fragmented nodes before and after treatment was plotted as a function of incubation time (Additional file 1: Fig. S3). MEFs wt kept the normal mitochondrial morphology after 72 h of treatment with lipoplexes. Again, this observation confirms the non-toxic character of these lipoplexes after long incubation times. In contrast, the incubation of OPA1-KO MEFs with transfection with (C_16_Im)_2_(C_4_O)/DOPE/DSPE-PEG/OPA1 lipoplexes at ρ_eff_ = 2.5 and 4 increased up to fourfold the ratio of elongated mitochondria after 8 h of treatment. The increase of the elongated fraction indicates a partial complementation of the normal phenotype by restoring the Opa1 fusion activity. As a control, the incubation of the ternary lipid mixture (without the OPA1 plasmid) did not produce any remodeling of the mitochondrial network either in MEFs wt or OPA1-KO MEFs (Additional file 1: Fig. S4). Note also that a similar transfection efficiency, as visualized by the complementation of the mitochondrial phenotype, was obtained with lipoplexes without PEGylated lipid and using a very similar plasmid in terms of effective charge and size [26]. This allows us to suggest that the transfection efficiency is, at least, not compromised by the presence of DSPE-PEG. Finally, we did not consider standard transfection reagents as a control due to the very low expression efficiency shown to complement the mitochondrial phenotype [26].

**Fig. 4:**
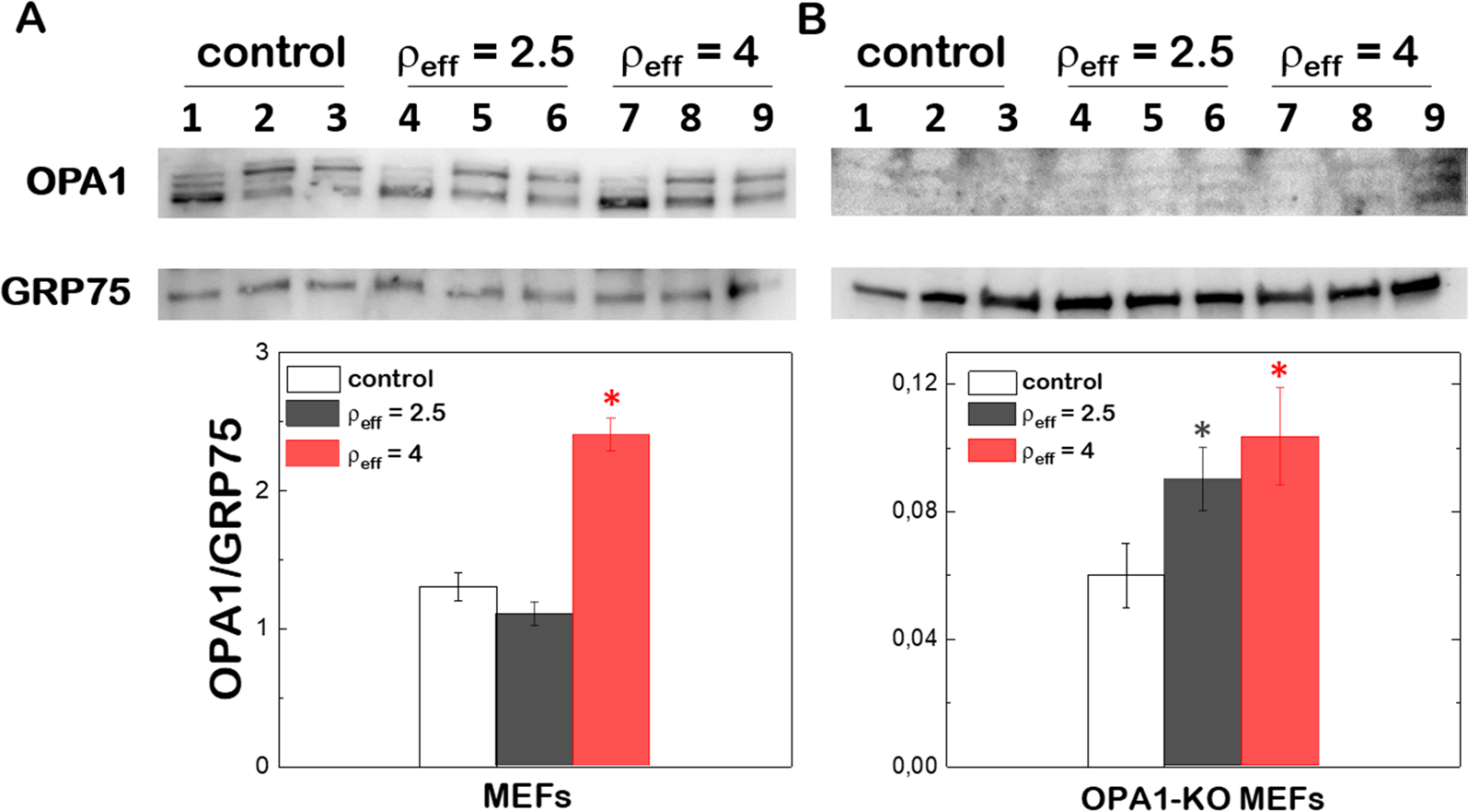
Western blot analysis of Opa1 and GRP75 protein levels in MEFs wt (**A**) and OPA1-KO MEFs (**B**) after transfection with (C_16_Im)_2_(C_4_O)/DOPE/DSPE-PEG/OPA1 lipoplexes at ρ_eff_ = 2.5 and 4 at different time intervals. The relative ratios of band intensity between Opa1 and GRP75 (lower panels) were quantified by ImageJ. Student’s t-test was performed to measure the significance of statistical difference between the different groups and the control (in the absence of treatment) from 3 independent experiments. (*p < 0.05 was considered statistically significant)

To unequivocally allocate the partial rescue of the normal mitochondrial phenotype to the presence of new copies of Opa1 protein after gene transfection, the expression levels of Opa1 were determined by immune-detection at different incubation times. The Opa1 levels were referred to GRP75 (OPA1/GRP75) or β-actin (OPA1/β-Actin) as house-keeping proteins to quantify the overexpression of exogenous Opa1 in MEFs wt and OPA1-KO MEFs (Fig. 4). Both human and murine cells produce up to 8 splicing variants (isoforms) of Opa1 [40, 41]. Isoforms are processed by at least three proteases forming long (L) and short (S) forms of Opa1 with different molecular sizes [42, 43]. L-forms are anchored to the IMM whereas S-forms are peripherally attached to the IMM with a fraction that diffuse in the Inter Membrane Space (IMS) [28, 40]. Western blots of mitochondrial lysates prepared from different tissues show a set of 5 bands (2 L-forms and 3 S-forms) with apparent molecular weights between 80 and 100 kDa with different relative abundance. In the particular case of MEFs, the lower band of the L-form and the two lower bands of S-forms are found to be more intense. As expected, we did not detect Opa1 in OPA1-KO MEFs, which results in mitochondrial fragmentation [44, 45].

We first quantified the Opa1 protein levels in MEFs wt treated with (C_16_Im)_2_(C_4_O)/DOPE/DSPE-PEG/OPA1 lipoplexes after 24 h of incubation and compared with the protein basal levels obtained in the absence of treatment (Fig. 4A). The densitometric analysis of western blots indicates no increase of the Opa1 protein levels after incubation with lipoplexes at ρ_eff_ = 2.5. However, we monitored a threefold increase of the Opa1 levels when cells were treated with lipoplexes at ρ_eff_ = 4. The absence of Opa1 basal levels in OPA1-KO MEFs allows us to allocate the protein overexpression directly to the action of lipoplexes (Fig. 4B). We detected the presence of three bands corresponding to one L-form and two S-forms. The densitometric analysis suggests that the protein levels of Opa1 after treatment did not reached similar values as those obtained for MEFs wt in the absence of treatment. We conclude that the mitochondrial network, completely fragmented in OPA1-KO-MEFs, is partially rescued by lipoplexes generating both L- and S-forms, in agreement with previous reports using viral carriers [46].

### Biodistribution and transfection efficiency of (C_16_Im)_2_(C_4_O)/DOPE/DSPE-PEG/OPA1 lipoplexes in mice

**Fig. 5:**
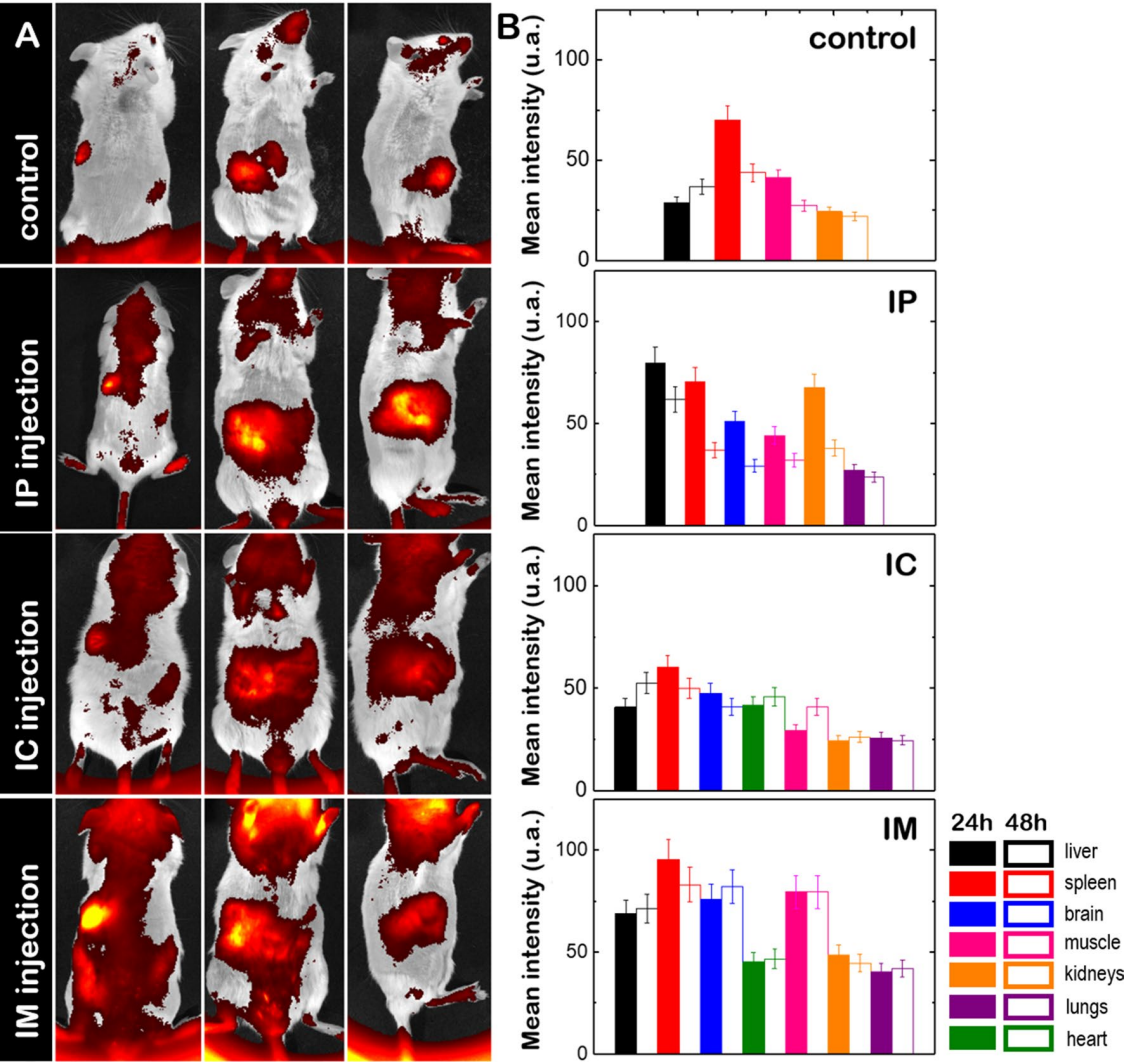
**A** In vivo biodistribution of DiR’-labeled (C_16_Im)_2_(C_4_O)/DOPE/DSPE-PEG/OPA1 lipoplexes in CD-1 mice after 48 h without treatment (control) and with treatment via intraperitoneal (IP), intracardiac (IC) and intramuscular (IM) administration. **B** Mean fluorescence intensity of ROIs enclosing each organ; (n = 1 animal per control and n = 3 animals per group)

The biodistribution of (C_16_Im)_2_(C_4_O)/DOPE/DSPE-PEG/OPA1 lipoplexes was traced by labeling the lipid mixture with DiR’. The DiR’-labeled lipoplexes (10 mg of mixed lipids/ kg of mouse) were administrated through different pathways (intraperitoneal, IP; intracardiac, IC and intramuscular, IM) and their accumulation in different organs was measured as the mean fluorescence intensity of ROIs enclosing the organs (Fig. 5A). We compared the biodistribution of lipoplexes after 24 h and 48 h of treatment with the basal intensity signal found in the absence of treatment. In the absence of treatment, a basal fluorescence intensity was found in vital organs like liver, spleen, kidneys and the muscle tissue from back legs (Fig. 5B). After IP administration the accumulation DiR-labeled lipoplexes was significantly increased in liver, spleen and kidneys but also lungs and brain were observed to accumulate lipoplexes (Fig. 5B). For an IC administration, the biodistribution pattern of lipoplexes was similar to that found in the absence of treatment, only the liver showed an increased accumulation as compared to control levels, and heart, brain and lungs were also labeled with DiR’, suggesting the bioaccumulation of lipoplexes in those organs (Fig. 5B). A general increased accumulation of lipoplexes was found after IM administration. Here, not only the fluorescent intensity levels found in liver, spleen, kidneys and muscles doubled their basal values but also the accumulation of lipoplexes in brain, heart and lungs raised when compared to other administration pathways (Fig. 5B). Our findings based on the comparative study of the three different injection routes allow us to establish the IM injection as the mouse preference-oriented drug administration. Independently of the administration pathway, we could also trace a progressive disappearance of lipoplexes within the mice after 4 weeks of incubation without producing any cytotoxicity to animals (Additional file 1: Fig. S5).

**Fig. 6:**
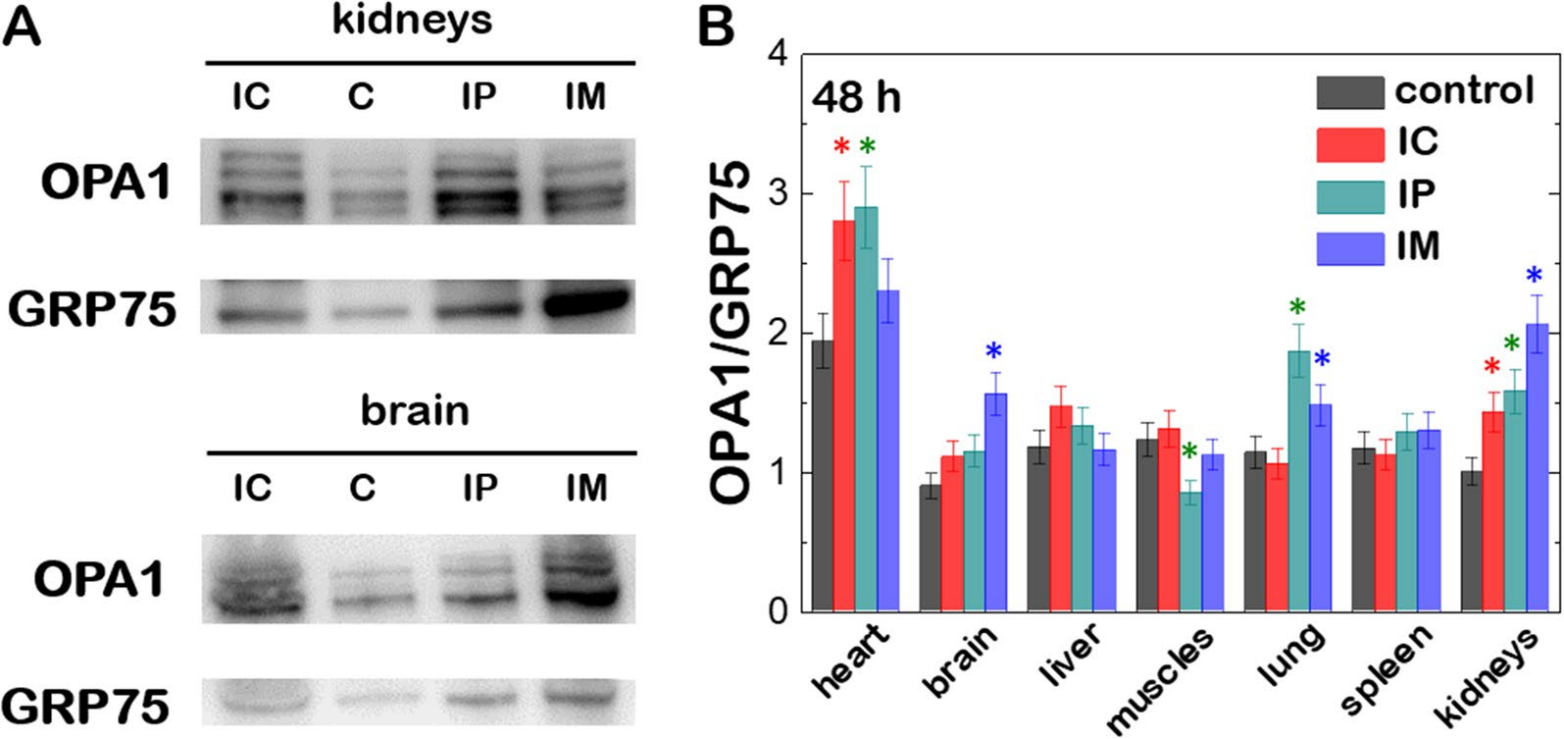
**A** Western blot of Opa1 and GRP75 protein levels in kidneys and brain from CD-1 mice after 48 h of transfection with (C_16_Im)_2_(C_4_O)/DOPE/DSPE-PEG/OPA1 lipoplexes (ρ_eff_ = 4). Different lanes correspond to the groups without treatment (C) and with treatment via intraperitoneal (IP), intracardiac (IC) and intramuscular (IM) administration. **B** Relative ratios of band intensity between Opa1 and GRP75 from different organs of CD-1 mice treated with (C_16_Im)_2_(C_4_O)/DOPE/DSPE-PEG/OPA1 lipoplexes (ρ_eff_ = 4) via intraperitoneal (IP), intracardiac (IC) and intramuscular (IM) administration. Results shown are mean ± s.e.m. from 3 independent experiments. The statistical significance was determined using a Student’s t-test between the different groups and the negative control (in the absence of treatment); (*p < 0.05 was considered statistically significant)

**Fig. 7.**
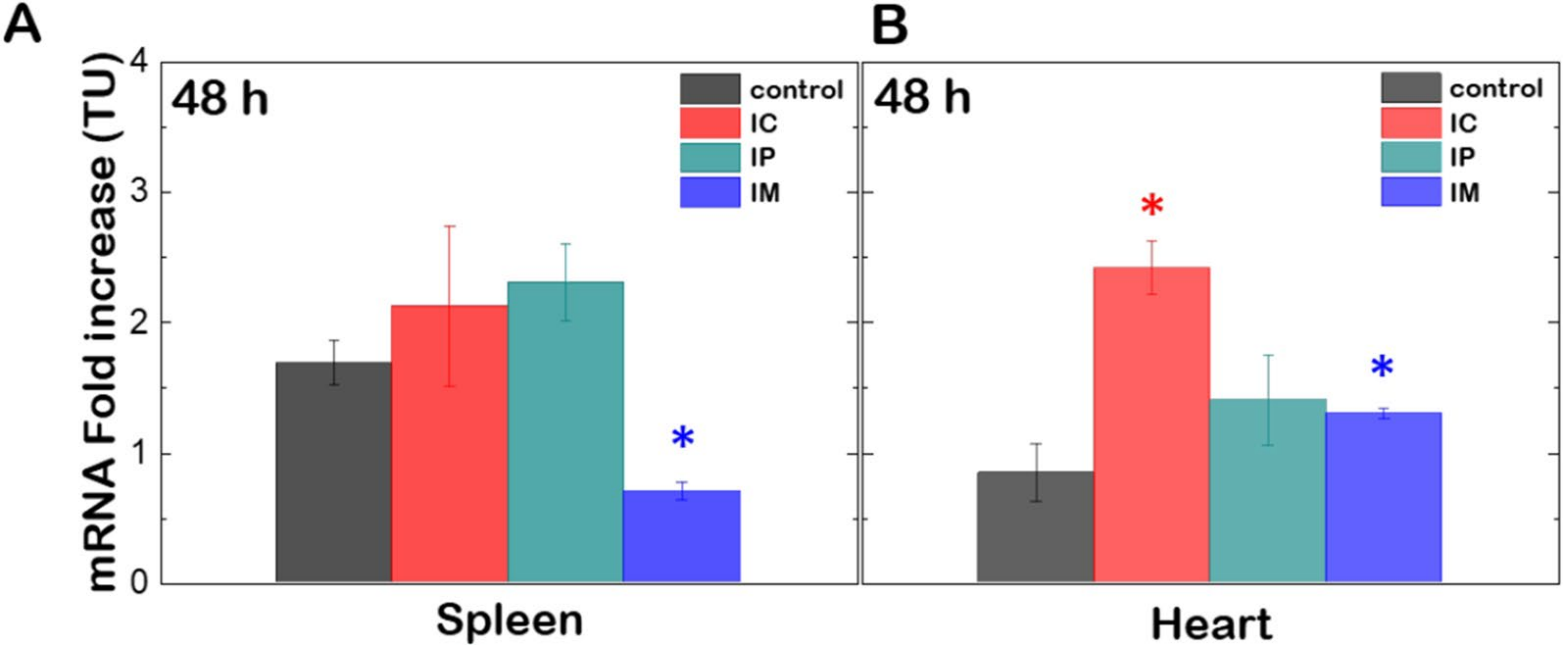
mRNA fold increase expression in spleen (**A**) and heart (**B**) from CD-1 mice after 48 h of transfection with (C_16_Im)_2_(C_4_O)/DOPE/DSPE-PEG/OPA1 lipoplexes (ρ_eff_ = 4). (*p < 0.05 was considered statistically significant).

We then evaluated the ability of (C_16_Im)_2_(C_4_O)/DOPE/DSPE-PEG/OPA1 lipoplexes to transport, deliver and overexpress Opa1 in mice. The Opa1 protein levels after 48 h incubation with lipoplexes at ρ_eff_ = 4 and administrated by either IP, IC or IM injection were compared to the protein background in different organs (heart, brain, lungs, muscles, liver, spleen and kidneys). After euthanasia, all organs presented normal coloration, texture and size after lipoplex treatment independently of the administration pathway. Western blots displayed different patterns containing up to 2 L-forms and 2 S-forms of Opa1 depending on the organ (Additional file 1: Fig. S6). After quantification, the densitometric analysis of western blots indicated an increase of ~ 50% of the Opa1 levels in heart, brain, lungs and kidneys (Fig. 6A). Independently of the administrations route, this is consistent with the onset of the accumulation of lipoplexes in brain and lungs and the increase of the fluorescence intensity in heart or kidneys after treatment (see Fig. 5). In contrast, the systematic higher accumulation of lipoplexes in the liver after administration did not result in a significant overexpression of Opa1 protein. A similar observation was made for spleen or muscles where the biodistribution of lipoplexes was not affected by IC or IP injections but highly increased by IM injection (Fig. 6B). The drug elimination function of the liver and spleen might explain the concomitant accumulation of lipoplexes in those organs without an efficient expression of the Opa1 protein though. A definitive evidence of Opa1 overexpression through lipoplexes was obtained by using RT-PCR, often considered as the gold standard to detect gene expression of a given transcript in a cell or tissue [47]. According to the western blot analysis shown in Fig. 6, heart and spleen were considered to be representative for an optimal and unresponsive Opa1 overexpression respectively, and were chosen for RT-PCR analysis. The data displays the fold change of the mRNA copies from the targeted gene expression (Fig. 7). We measured a 50–100% increase of the mRNA-fold in heart after 48 h incubation with lipoplexes at ρ_eff_ = 4. In contrast, the same treatment did not result in any change of the mRNA fold increase in spleen. These results were consistent with the higher accumulation and overexpression of Opa1 in heart as measured by in vivo imaging experiments (Fig. 5) and western blotting (Fig. 6).

Face to viral vectors, lipofection represents less than 5% of the ongoing clinical trials based on gene therapy [48]. The main reason for this evidence lies on the lower transfection efficiency and the unfavorable biodistribution of lipoplexes relative to viral vectors [49]. Despite of the benefits of using lipid/DNA complexes associated to their safety (non-toxicity and non-immunogenicity), their versatile formulation and their easy manufacture; the first generation of cationic lipids displayed a very dissimilar biodistribution and transfection efficiency. Despite some authors reported a successful in vivo transfection with an accumulation of lipoplexes in lung, liver, spleen, heart and kidneys, lasting for several days or weeks [50–54] other reports signaled a low activity of expressed proteins in systemic organs [55–57] or a maximal protein activity after 24 h of administration [58, 59]. The observed discrepancy between reports might lay on the different promoters, reporter genes, or the detection method used. However, the intravenous administration used in those reports seems to show a similar biodistribution pattern that consists on a rapid accumulation in lungs followed by a hepatic clearance [60]. The strong interaction between cationic lipids and the bloodstream proteins mediates the rapid elimination of lipoplexes through the RES [61].

Although the contribution of the PEG moiety to biodistribution is not straightforward, since no controls in the absence of the PEGylated lipid was carried out, a few studies report the biodistribution of lipoplexes based on gemini-cationic lipid with disparate results. Whereas local administration (topic and ocular) produced significantly high expression of the reporter plasmids [62, 63], intravenous administration through the penile vein of pH-sensitive sugar-based gemini cationic lipids showed a local accumulation at the site of injection and a remarkable expression of the protein only in liver and spleen [64]. Intravenous administration of pH-sensitive sugar-based gemini cationic lipids trough the tail vein leaded to a transfection efficiency that was sevenfold higher than the commercially available lipofection agents in lung and heart after 24 h of treatment [65]. However, the intravenous administration through the tail vein of quaternary gemini cationic lipids-based lipoplexes resulted in their accumulation mainly in liver and spleen for 24 h that precedes a first accumulation in lungs within the first minutes after injection [16]. Again, the rapid redistribution of lipoplexes from lungs to vital organs for drug elimination such as liver, spleen or kidneys is observed upon an intravenous administration.

Here, we show that the specific use of a gemini cationic lipid combined with a PEGylated lipid does not completely avoid the preliminary capture of lipoplexes in the lungs but allows for a wide and sustained biodistribution of lipoplexes in vital organs such as heart or brain after IM administration. The delocalized positive charge of gemini cationic lipids [24], the furtiveness of lipoplexes provided by the polymer coating [18–20] and the local injection might hamper the electrostatic interactions of cationic surfactants with the plasma proteins and the following rapid clearance of lipoplexes by the RES. Moreover, the overexpression of the Opa1 protein in those organs was mostly enhanced after treatment (Fig. 6). This observation is particularly interesting as a high increase of Opa1 levels might protect from heart and brain damage induced by ischemia–reperfusion injury [66]. Likewise, the specific targeting of gemini cationic lipid-based lipoplexes to skeletal muscle may be further exploited given that myopathy is one of the most frequent clinical symptoms of ADOA. Although we did not detect a significant increase in the Opa1 levels in muscles, we demonstrate a local accumulation of lipoplexes in that tissue. However, two major drawbacks have been identified for an optimal OPA1-gene therapy [67]. First, the slight overexpression of Opa1 in mice upon transfection with viral nanocarriers [66] and second, the inability of single Opa1 isoforms to completely recover the network dynamics [68]. The use of cubic gemini cationic lipid-based nanovectors might represent an alternative to viral vectors and overcome the stated issues. Additional work is required to explore (a) the simultaneous transfection of multiple plasmids coding for different Opa1 isoforms and (b) the local administration of lipoplexes within eye, as this organ has been shown to be an excellent target for mitochondrial optic neuropathies gene therapy [69, 70].

## Conclusions

Despite the growing knowledge on the physicochemical properties of lipoplexes and the development of versatile formulations displaying high transfection yields in living cells, the extensive use of lipofection in gene therapy is still in progress. Non-viral gene therapy clinical trials targeting multiple diseases have increased in the last decade, but only 30% of them are based on lipid nanocarriers [71]. In particular, gemini cationic lipids have not been explored yet in clinical trials and a gemini cationic lipid-based gene therapy remains elusive due to the inconclusive demonstration of an efficient and specific transgene delivery. We show that (C_16_Im)_2_(C_4_O), along with DOPE and a PEGylated lipid, in conjunction with plasmid DNA can form bicontinuous cubic lipoplexes able to transport and release a plasmid coding for the Opa1 mitochondrial protein in living cells. Upon treatment, the fragmented mitochondrial phenotype observed in OPA1-KO MEFs is partially recovered and accompanied with an overexpression of the Opa1 protein. Moreover, the Opa1 protein was overexpressed in vital organs such as heart or brain when lipoplexes were administrated into mice through intramuscular pathway. Our results pave the way for using synthetic gemini cationic lipid-based cationic lipoplexes as therapeutic non-viral gene nanocarriers against mitochondrial dysfunctions.

## Methods

### Lipids and plasmids

The gemini cationic lipid bis (hexadecyl dimethyl imidazolium) oxyethylene (C_16_Im)_2_(C_4_O), with a polyoxythelyne spacer of one oxygen and four carbon atoms (Scheme S1), was synthesized according to ref [72]. The zwitterionic phospholipid 1,2-dioleoyl-sn-glycerol-3-phosphoethanolamine (DOPE) and 1,2-distearoyl-sn-glycerol – 3 – phospho ethanolamine-N-[carboxy(polyethylene glycol)-2000] (sodium salt) (DSPE-PEG2000) (DSPE-PEG) were purchased from Avanti Polar Lipids (Additional file 1: Scheme S1). The fluorescent dye 1,1'-Dioctadecyl-3,3,3',3'-Tetramethylindotricarbocyanine Iodide (Invitrogen™ DiR') was provided by Fisher Scientific. The plasmids pMSCV-OPA1 encoding a variant for the OPA1 human transcript splice form 1 were synthesized according to ref. [31].

### Chemicals and antibodies

N-2-hydroxyethylpiperazine-N′-2-ethanesulfonic acid (HEPES), HEPES (sodium salt), HEPES (free acid), Tetramethylrhodamine (TMRM, T668), the AlamarBlue cell viability reagent (DAL1025), the NUPAGE LDS sample buffer (4×) from Novex, the Complete Mini Protease Inhibitor cocktail from Roche Applied Sciences and the BCA Assay Kit from Pierce, the RIPA lysis buffer 10× from Millipore were all provided by Fisher Scientific. Lipofectamine2000 Transfection reagent (11668019) was purchased from Thermo Fisher Scientific. Rhodamine123 (R8004), LysoTracker Green (DND-26), DL-dithiothreitol (DTT; D0632) and β-mercaptoethanol were purchased from Sigma Aldrich. Mouse Anti-Opa1 monoclonal antibody (1:2000) was purchased by BD Biosciences (#612607). β-actin (1:2000) and GRP-75 (1:2000) antibodies were purchased from Santa Cruz Biotechnology (#sc-47778 and SC-133137, respectively). Anti-mouse and anti-rabbit HRP secondary antibodies were provided by GE Healthcare (#GENA931 and A0545, respectively). GAPDH, glyceraldehyde-3-phosphate dehydrogenase (ab8245), was purchased from Abcam.

### Mixed lipids and lipoplexes preparation

A dry film of (C_16_Im)_2_(C_4_O), DOPE and DSPE-PEG at a molar ratio of 15/80/5 respectively (15/80/4/1.3 for (C_16_Im)_2_(C_4_O)/DOPE/DSPE-PEG/DiR’) was prepared after removing the chloroform solvent under high vacuum. The lipid film (5 mg/mL final concentration) was then hydrated with 40 mM HEPES buffer at a pH = 7.4 and sequentially extruded to a final diameter of 100 nm [73]. Lipoplexes were formed at 37ºC by mixing different amounts of MSCV-OPA1 plasmid with (C_16_Im)_2_(C_4_O)/DOPE/DSPE-PEG mixed lipids for 30 min, expressed in effective charge ratio (ρ_eff_) between ± charge ratio of (C_16_Im)_2_(C_4_O)/DOPE/DSPE-PEG mixed lipids and DNA.

### Zeta potential

(C_16_Im)_2_(C_4_O)/DOPE/DSPE-PEG mixed lipids and lipoplexes were prepared with 40 mM HEPES at pH = 7.4. The concentration of the MSCV-OPA1 plasmid was kept as 0.05 mg/mL varying the concentration of the (C_16_Im)_2_(C_4_O)/DOPE/DSPE-PEG mixed lipids to produce different m_L_/m_D_ mass ratios. Electrophoretic mobility of (C_16_Im)_2_(C_4_O)/DOPE/DSPE-PEG/OPA1 lipoplexes was measured, at 25ºC, as a function of the m_L_/m_D_ mass ratio on a Zeta Potential Analyzer (Brookhaven Instrum. Corp., USA). Each electrophoretic mobility value was taken as an average of 30 independent measurements. Zeta potential ($\zeta$) was obtained from the electrophoretic mobility (${\mu }_{e}$) using the Henry equation

1 $$\zeta = \frac{3\eta {\mu }_{e}}{2{\varepsilon }_{0}{\varepsilon }_{r}f({\kappa }_{D}a)}$$

where $\eta$ is the water viscosity (8.904 × 10^−4^ N m^−2^ s at 298.15 K); ${\varepsilon }_{0}$ and ${\varepsilon }_{r}$ are the vacuum and relative permittivity (8.854 × 10^−12^ J^−1^·C^2^·m^−1^ and 78.5, respectively); and $f({\kappa }_{D}a)$ the Henry function which depends on the reciprocal Debye length (${\kappa }_{D}$) and the hydrodynamic particle radius ($a$). For medium-to-large particles in a medium of moderate ionic strength ($a\gg {\left({\kappa }_{D}\right)}^{-1}$), $f({\kappa }_{D}a)$ is assumed as 1.5 following the Smoluchowski approximation [74, 75].

### Small angle x-ray scattering (SAXS)

SAXS experiments were carried out in the beamline NCD11 at the ALBA Synchrotron (Barcelona, Spain). The energy of the incident beam was 12.6 keV (λ = 0.9998 Å). Samples were placed in sealed glass capillaries. The scattered X-rays were detected on a Quantum 210r CCD detector, converted to one-dimensional scattering by radial averaging, and represented as a function of the momentum transfer vector (q, nm^−1^). The sample to detector distance was of 2.88 m. SAXS experiments were performed for (C_16_Im)_2_(C_4_O)/DOPE/DSPE-PEG/OPA1 lipoplexes at effective charge ratio ρ_eff_ = 2.5 and 4. In each capillary, the amount of MSCV-OPA1 was 9 µg and the amount of (C_16_Im)_2_(C_4_O)/DOPE/DSPE-PEG mixed lipid was changed accordingly keeping a final volume of 80 µL/capillary. Measurements at each lipoplex composition were run by duplicate in two independent capillaries.

### Cell cultures

Mouse embryonic fibroblasts (MEF wt) and *OPA1*-Knockout fibroblasts were from ATCC (CRL-2991 and CRL-2995 respectively). Cells were cultured in complete Dulbecco Modified Eagle Medium (DMEM) supplemented with 10% Premium Fetal bovine serum (FBS, South Africa S1300; Biowest, Nuallé, France), 1% non-essential amino acids (MEM-NEA), 1% penicillin/Streptomycin (final concentration of 100 U/mL of penicillin and 100 μg/mL of streptomycin), all purchased from Gibco. The cells were grown in a humidified incubator (Forma Steri-Cycle ThermoFisher with HEPA filter, 5% CO_2_) at 37 °C and maintained with a split ratio of 1:15 at 80% of confluence in T75 flasks (Nunc). Trypsine/EDTA 0.05% from Hyclone was used to detach cell cultures from flasks. Dulbecco Phosphate Buffered Saline without calcium and magnesium (DPBS) from Gibco and Phosphate Buffered Saline 1x (PBS) from Hyclone, were used for washing cells.

### Confocal laser scanning microscopy (CLSM)

The cellular uptake of lipoplexes and the mitochondrial network of MEFS and OPA1-KO MEFs were monitored using a confocal Leica TCS SP5 inverted microscope with a near-IR laser (750 nm) and a green laser (488 nm) or an inverted Nikon Ti-E microscope equipped with a Nikon scanning confocal microscope module C2, Nikon Plan Apo 100 × NA 1.45 oil immersion objective and two lasers (488 and 561 nm; Sapphire laser). 10^5^ cells per cm^2^ of MEFs wt or *OPA1*-Knockout MEFs in a 4-well Nunc™ Lab-Tek™ slide (Thermo Fisher) containing 500 μL of complete DMEM were treated with lipoplexes. Lipoplexes were prepared using MSCV-OPA1 and diluted with complete DMEM at a final concentration of 0.36 µM per well and at different ρ_eff_ of 2.5 or 4. For the mitochondrial morphology experiments, cells were labeled with Rhodamine123 (5.3 µM) or TMRM (1 µM) 30 min before confocal imaging at different time intervals (0, 24 h, 48 h and 72 h). For a given picture, the statistical distribution of both the elongated (including the branched nodes or mitochondrial networks) and fragmented non-branched nodes (individual round mitochondria) were determined by using the Skeleton Analyzer plug-in for ImageJ [27, 41]. For each condition, the analysis was made for a population of three independent samples comprising more than 1000 ± 200 different mitochondria. The time evolution of the mitochondrial network was determined as the % of elongated mitochondria normalized by the initial % of elongated mitochondria before treatment. To follow the uptake of lipoplexes inside MEFs wt and OPA1-KO MEFs, lipoplexes were labeled with DiR’ (1.3% mol of the lipid composition). Prior to lipoplex incubation, MEFs wt and OPA1-KO MEFs were labeled with Lysotracker Green (1 µM). Cells were then imaged at different periods of time. The overlap of the DiR’ and Lysotracker signals was taken as indicative for a colocalization between lipoplexes and lysosomes. An additional proof for the cellular uptake of the mixed lipids labeled with DiR’ was obtained on a Cytell Cell Imaging System (GE Healthcare Life Sciences). Here, 10^5^ cells/well of MEFs wt were seeded in a 6-well plate. After 24 h, cells were transfected with mixed lipids at different concentrations and the localization was monitored after 24 h (see Additional file 1: Figure S1).

### Cell viability assays

Cell viability was evaluated by the alamarBlue assay (ThermoFisher) following the manufacturer’s protocol. 1000 cells were seeded in a 96-well plate and grown up to ~ 80% confluence (at 37 °C, 5% CO_2_, 99% humidity). After incubation with lipoplexes (0.1 µg of MSCV-OPA1 for 6 h, cells were washed with DPBS and re-incubated with complete growth medium (DMEM with 10% FBS without Red Phenol) for different periods of time. 10% of alamarBlue was added two hours before measuring the absorbance on a Multiskan FC plate reader (ThermoFisher scientific). The absorbance at 570 nm was monitored using 620 nm as the reference wavelength. The viability was determined by comparison with control cells in the absence of treatment (100%). Lipofectamine2000 was used as a positive control following the manufacturer’s protocol. All reported experiments were performed in triplicate. Alternatively, cell viability was also monitored with the Annexin-V assay. 10^5^ MEFs wt and *OPA1*-KO MEFs with and without treatment with lipoplexes (0.36 µg of MSCV-OPA1 at a charge ratio ρ_eff_ = 2.5 and 4) were seeded in 12-well plates. After 24 h, cells were stained with Annexin V-Alexa568 (Roche) according to manufacturer`s protocol. Apoptosis levels were measured by flow cytometry (FACS Calibur BD Biosciences) as the percentage of Annexin-V positive events of the total population.

### Biodistribution studies

Biodistribution studies of DiR’-labeled lipoplexes were carried out according to approved protocols (protocol 32/2011 CEASA University of Padua and 318/2015 Italian Ministry of Health to L.S). 10 male non-consanguineous CD-1 mice of 12 weeks old were maintained in a temperature (22ºC) and humidity (30–50%) controlled animal care facility with a 12 h light/dark cycle and free access to water and food. After intraperitoneal (N = 3), intracardiac (N = 3) or intramuscular administration (N = 3) of DiR’-labeled lipoplexes (400 µg/per animal), mice were anesthetized with a mixed solution composed of 15 µL of rompun (Xylazine, 20 mg/mL) and 25 µL of virbac zoletil (Tiletamine/zolazepam, 10/10 mg/mL). The biodistribution of DiR- labeled lipoplexes at different time intervals, was then determined on a Xenogen IVIS-100 (Perkin-Elmer) using the Cy5.5 filter (675/694 nm). After treatment, animals were sacrificed in a CO_2_ chamber with gradual CO_2_ and O_2_ flux and by cervical dislocation. Subsequently, tissues from brain, spleen, liver, lungs, kidney, muscle and heart were dissected, washed with saline solution and frozen in liquid nitrogen for Western Blot analysis and RT-PCR.

### Western blot analysis

For in vitro experiments, 5 × 10^5^ cells/well of MEFs wt and OPA1-KO MEFs with and without transfected lipoplexes were seeded in 6-well plate and incubated for 24, 48 and 72 h. Cells were then harvested by centrifugation for 1 min at 2544 g (Thermo Scientific Sorvall ST 8 Centrifuge). After washing with PBS (1 min at 2544 g) cells were lysed in 50 µL of lysis buffer (1 × RIPA complemented with Complete Mini Protease Inhibitor cocktail) for 30 min in ice. The supernatant with proteins was collected after centrifugation for 10 min at 18,000 g and 4ºC. Protein concentration was determined by BCA Assay using a Genesys 10S UV–vis Spectrophotometer (ThermoFisher Scientific). Proteins were then heated to 70ºC for 10 min in NUPAGE LDS sample buffer (4 ×) supplemented with 5% of β-mercaptoethanol. 20 μg of total protein was loaded per lane on a 7.5% SDS-polyacrylamide gel. SDS-PAGE and western blotting were performed according to standard procedures with NUPAGE LDS sample buffer, polyvinylidene difluoride Western Blotting membrane**s** (Roche), anti-OPA1 (1:2000 dilution), anti-GRP75 (1:2000 dilution), β-actin (1:2000 dilution) and secondary HRP antibodies (1:2000 dilution) for the detection of specific proteins. Bands were developed with Luminata chemiluminescent HRP substrate (Merck Millipore) on a ImageQuant LAS 4000 (GE Healthcare). ImageJ was used for protein level quantification by densitometric analysis. For in vivo experiments, frozen mouse tissues were lysed with 200-500 µL of lysis buffer (1 × RIPA complemented with Complete Mini Protease Inhibitor cocktail) for 2 cycles of 30 s at 30 Hz on a TissueLyser II (QUIAGEN). After incubation for 30 min on ice, the mitochondrial- enriched fraction was collected by centrifugation (15,429 g for 10 min at 4ºC in the presence of protease inhibitors). Protein concentration was determined by BCA Assay. SDS-PAGE and western blotting were performed as for in vitro experiments.

### RT-PCR

For the analysis of transcripts, total RNA was extracted from liquid-nitrogen snap-frozen spleen and heart specimens with guanidinium thiocyanate, according to manufacturer instructions (TRIzol, Invitrogen, Carlsbad, CA, USA). Tissue Lyser II (30 Hz 1 min (× 2)) was used to help organs to lysate. 400 ng of total RNA were incubated first with random primer hexamers and dntPs for 5 min at 65 °C in a total volume of 13 µL. Then, RNA was retro-transcribed with SuperScript IV reverse transcriptase (Life Technologies) using RNase Out (Life technologies), DTT and SupersCript IV in a final volume of 20 µL. The used reaction program was: 23 °C 10 min, 55 °C 10 min and 80° 10 min. 8 ng of cDNA was amplified by RT-PCR assay using SYBR Green chemistry (Applied Biosystems) and primers specifics for the amplification of OPA1 and for actin and GAPDH as house-keeping genes were designed. Fold change values were calculated using the ΔΔCt method and an unpaired t-test was used to calculate statistical significance.

### Statistical analysis

Student’s t-test was performed to measure the significance of statistical difference between the different groups and the negative control (in the absence of treatment). Differences were considered statistically significant for p < 0.05.

## Supplementary Information

**Additional file 1.** Supporting material.

## Data Availability

All data generated or analyzed during this study are included in this published article.

## Abbreviations

ADOA: Autosomal Dominant Optic Atrophy
DOPE: 1,2-Dioleoyl-sn-glycero-3-phosphoethanolamine
(C_16_Im)_2_(C_4_O): Gemini cationic lipids
CD-1: Crl:CD1(ICR) white Outbred Swiss mice
CLSM: Confocal Laser Scanning Microscopy
ctDNA: Calf-thymus DNA
DiR: Fluorescent dye 1,1'-Dioctadecyl-3,3,3',3'-Tetramethylindotricarbocyanine Iodide
DMEM: Dulbecco Modified Eagle Medium
DNA: Deoxyribonucleic acid
DOPE: 1,2-Dioleoyl-sn-glycerol-3-phosphoethanolamine
DPBS: Dulbecco Phosphate Buffered Saline
DSPE-PEG: 1,2-Distearoyl-sn-glycero-3-phosphoethanolamine-N-[carboxy(polyethylene glycol)-2000]
DTT: DL-dithiothreitol
FBS: Fetal Bovine Serum
GAPDH: Glyceraldehyde-3-phosphate dehydrogenase
GFP: Green Fluorescence Protein
GRP75: 75 KDa glucose-regulated protein
GTP: Guanosine triphosphate
HEPES: (4-(2-Hydroxyethyl)-1-piperazineethanesulfonic acid
IC: Intracardiac
IM: Intramuscular
IMM: Inner Mitochondrial Membrane
IMS: Inter Membrane Space
IP: Intraperitoneal
MEFs: Mouse Embryonic Fibroblasts
MOG: Monoolein glycerol
MSCV-OPA1: Plasmid DNA coding for Opa1 protein
OPA1: Optic atrophy type 1 (autosomal dominant), Mitochondrial Dynamin Like GTPase
OPA1-KO MEFs: Mouse embryonic Fibroblasts Knockout in OPA1 protein
pDNA: Plasmid deoxyribonucleic acid
PBS: Phosphate Buffer Saline
RES: Reticuloendothelial system
RNA: Ribonucleic acid
RT-PCR: Reverse Transcription Polymerase Chain Reaction
SAXS: Small angle X-ray scattering
TMRM: Tetramethylrhodamine
WB: Western Blots

## References

[1] Dunbar CE, High KA, Joung JK, Kohn DB, Ozawa K, Sadelain M (2018). Gene therapy comes of age. Science.

[2] Safinya CR, Ewert K, Ahmad A, Evans HM, Raviv U, Needleman DJ, Lin AJ, Slack NL, George C, Samuel CE (2006). Cationic liposome–nucleic acid complexes for gene delivery and gene silencing. Philos Trans R Soc A.

[3] Luten J, van Nostrum CF, De Smedt SC, Hennink WE (2008). Biodegradable polymers as non-viral carriers for plasmid DNA delivery. J Controlled Release.

[4] Felgner JH, Gadek TR, Holm M, Roman R, Chan HW, Wenz M, Northrop JP, Ringold GM, Danielsen M (1987). Proc Natl Acad Sci USA.

[5] Radler JO, Koltover I, Salditt T, Safinya CR (1997). Science.

[6] Koltover I, Salditt T, Rädler JO, Safinya CR (1998). An inverted hexagonal phase of cationic liposome–DNA complexes related to DNA release and delivery. Science.

[7] Junquera E, Aicart E (2016). Recent progress in gene therapy to deliver nucleic acids with multivalent cationic vectors. Adv Colloid Interface Sci.

[8] de la Fuente-Herreruela D, Monnappa AK, Muñoz-Úbeda M, Morallón-Piña A, Enciso E, Sánchez L, Giusti F, Natale P, López-Montero I (2019). Lipid–peptide bioconjugation through pyridyl disulfide reaction chemistry and its application in cell targeting and drug delivery. J Nanobiotechnol.

[9] Farhood H, Serbina N, Huang L (1995). The role of dioleoyl phosphatidylethanolamine in cationic liposome mediated gene transfer. Biochim Biophys Acta.

[10] Rappolt M, Hickel A, Bringezu F, Lohner K (2003). Mechanism of the lamelar/inverse hexagonal phase transition examined by high resolution X-ray diffraction. Biophys J.

[11] Sarkar S, Tran N, Rashid MH, Le TC, Yarovsky I, Conn CE, Drummond CJ (2019). Toward cell membrane biomimetic lipidic cubic phases: a high-throughput exploration of lipid compositional space. ACS Appl Bio Mater.

[12] Leal C, Bouxsein NF, Ewert KK, Safinya CR (2010). Highly efficient gene silencing activity of siRNA embedded in a nanostructured gyroid cubic lipid matrix. J Am Chem Soc.

[13] Martínez-Negro M, Kumar K, Barrán-Berdón AL, Datta S, Kondaiah P, Junquera E, Bhattacharya S, Aicart E (2016). Efficient cellular knockdown mediated by siRNA nanovectors of gemini cationic lipids having delocalizable headgroups and oligo-oxyethylene spacers. ACS Appl Mater Interfaces.

[14] Damen M, Groenen AJJ, van Dongen SFM, Nolte RJM, Scholte BJ, Feiters MC (2018). Transfection by cationic gemini lipids and surfactants. Med Chem Comm.

[15] Juliano RL (1988). Factors affecting the clearance kinetics and tissue distribution of liposomes, microspheres and emulsions. Adv Drug Deliv Rev.

[16] Yadav MR, Kumar M, Murumkar PR, Hazari PP, Mishra AK (2018). Gemini amphiphile-based lipoplexes for efficient gene delivery: synthesis, formulation development, characterization, gene transfection, and biodistribution studies. ACS Omega.

[17] Allen TM, Hansen C, Martin F, Redemann C, Yau-Young A (1991). Liposomes containing synthetic lipid derivatives of poly(ethylene glycol) show prolonged circulation half-lives in vivo. Biochim Biophys Acta.

[18] Bai J, Liu Y, Sun W, Chen J, Miller AD, Xu Y (2013). Down-regulated lysosomal processing improved pegylated lipopolyplex-mediated gene transfection. J Gene Med.

[19] Gjetting T, Arildsen NS, Christensen CL, Poulsen TT, Roth JA, Handlos VN, Poulsen HS (2010). In Vitro and in Vivo Effects of Polyethylene Glycol (PEG)-modified Lipid in DOTAP/cholesterol-mediated Gene Transfection. Int J Nanomedicine.

[20] Bombelli C, Faggioli F, Luciani P, Mancini G, Sacco MG (2007). PEGylated lipoplexes: preparation protocols affecting DNA condensation and cell transfection efficiency. J Med Chem.

[21] Muñoz-Úbeda M, Misra SK, Barrán-Berdón AL, Aicart-Ramos C, Sierra MB, Biswas J, Kondaiah P, Junquera E, Bhattacharya S, Aicart E (2011). Why is less cationic lipid required to prepare lipoplexes from plasmid DNA than linear DNA in gene therapy?. J Am Chem Soc.

[22] Muñoz-Úbeda M, Misra SK (2012). How does the spacer length of cationic gemini lipids influence the lipoplex formation with plasmid DNA? Physicochemical and biochemical characterizations and their relevance in gene therapy. Biomacromol.

[23] Martínez-Negro M, Kumar K, Barrán-Berdón AL, Datta S, Kondaiah P, Junquera E, Bhattacharya S, Aicart E (2016). Efficient cellular knockdown mediated by siRNA nanovectors of gemini cationic lipids having delocalizable headgroups and oligooxyethylene spacers. ACS Appl Mater Interfaces.

[24] Kumar K, Barrán-Berdón AL, Datta S, Muñoz-Úbeda M (2015). A delocalizable cationic headgroup together with an oligooxyethylene spacer in gemini cationic lipids improves their biological activity as vectors of plasmid DNA. J Mater Chem B.

[25] Misra SK, Muñoz-Úbeda M, Datta S, Barrán-Berdón AL (2013). Effects of a delocalizable cation on the headgroup of gemini lipids on the Lipoplex-type nanoaggregates directly formed from plasmid DNA. Biomacromol.

[26] Muñoz-Úbeda M, Tolosa-Díaz A, Bhattacharya S, Junquera E, Aicart E, Natale P, López-Montero I (2019). Gemini-based lipoplexes complement the mitocondrial phenotype in MFN1-Knockout Fibroblasts. Mol Pharm.

[27] Chan DC (2006). Mitochondrial fusion and fission in mammals. Annu Rev Cell Dev Biol.

[28] Olichon A, Emorine LJ, Descoins E, Pelloquin L, Brichese L, Luptak I, Guillou E, Delettre C, Valette A, Hamel CP (2002). The human dynamin—related protein OPA1 is anchored to the mitochondrial inner membrane facing the inner-membrane space. FEBS Lett.

[29] Delettre C, Lenaers G, Griffoin JM, Gigarel N, Lorenzo C, Belenguer P, Pelloquin L, Grosgeorge J, Turc-Carel C, Perret E (2000). Nuclear gene OPA1, encoding a mitochondrial dynamin-related protein, is mutated in dominant optic atrophy. Nat Genet.

[30] Cipolat S, Martins de Brito O, Dal Zilio B, Scorrano L. OPA1 requires mitofusin 1 to promote mitochondrial fusion. Proc Natl Acad Sci USA. 2004; 101: 15927–15932. 10.1073/pnas.0407043101 PMC528769 15509649

[31] Detmer SA, Chan DC (2007). Complementation between mouse Mfn1 and Mfn2 protects mitochondrial fusion defects caused by CMT2A disease mutations. J Cell Biol.

[32] Cardoso AM, Morais CM, Cruz AR, Cardoso AL, Silva SG, Luisa M, Marques EF, Pedroso MC, Jurado AS (2015). Gemini surfactants mediate efficient mitochondrial gene delivery and expression. Mol Pharm.

[33] Lin AJ, Slack NL, Ahmad A, George CX, Samuel CE, Safinya CR (2003). Three-dimensional imaging of lipid gene-carriers: membrane charge density controls universal transfection behavior in lamellar cationic liposome-dna complexes. Biophys J.

[34] Mezzenga R, Meyer C, Servais C, Romoscanu AI, Sagalowicz L, Hayward RC (2005). Shear rheology of lyotropic liquid crystals: a case study. Langmuir.

[35] Leung SSW, Leal C (2019). The stabilization of primitive bicontinuous cubic phases with tunable swelling over a wide composition range. Soft Matter.

[36] Briggs J, Chung H, Caffrey M (1996). The temperature-composition phase diagram and mesophase structure characterization of the monoolein/water system. J Phys.

[37] Tyler AII, Barriga HMG, Parsons ES, McCarthy NLC, Ces O, Law RV, Seddon JM, Brooks NJ (2015). Electrostatic swelling of bicontinuous cubic lipid phases. Soft Matter.

[38] Bilalov A, Olsson U, Lindman B (2009). A cubic DNA- Lipid complex. Soft Matter.

[39] Valente AJ, Maddalena LA, Robb EL, Moradi F, Stuart JA (2017). A simple ImageJ macro tool for analyzing mitochondrial network morphology in mammalian cell culture. Acta Histochemia.

[40] Delettre C, Griffoin JM, Kaplan J, Dolfus H, Lorenz B, Faivre L, Lenaers G, Belenguer P, Hamel CP (2001). Mutation spectrum and splicing variants in the OPA1 gene. Hum Genet.

[41] Akepati VR, Müller EC, Otto A, Strauss HM, Portwich M, Alexander C (2008). Characterization of OPA1 isoforms isolated from mouse tissues. J Neurochem.

[42] Song Z, Chen H, Fiket M, Alexander C, Chan DC (2007). OPA1 processing controls mitochondrial fusion and is regulated by mRNA splicing, membrane potential, and Yme1L. J Cell Biol.

[43] Anand R, Wai T, Baker MJ, Kladt N, Schauss AC, Rugarli E, Langer T (2014). The i-AAA protease YME1L and OMA1 cleave OPA1 to balance mitochondrial fusion and fission. J Cell Biol.

[44] Lee H, Smith SB, Yoon Y (2017). The short variant of the mitochondrial dynamin OPA1 maintains mitochondrial energetics and cristae structure. J Biol Chem.

[45] Patten DA, Wong J, Khacho M, Soubannier V, Mailloux RJ, Pilon-Larose K, MacLaurin JG, Park DS, McBride HM, Trinkle-Mulcahy L, Harper ME, Germain M, Slack RS (2014). OPA1-dependent cristae modulation is essential for cellular adaptation to metabolic demand. EMBO.

[46] Del Dotto V, Mishra P, Vidoni S, Fogazza M, Maresca A, Caporali L, McCaffery JM, Cappelletti M, Baruffini E, Lenaers G, Chan D, Rugolo M, Carelli V, Zanna C (2017). OPA1 isoforms in the hierarchical organization of mitochondrial functions. Cell Rep.

[47] Nolan T, Hands RE, Bustin SA (2006). Quantification of mRNA using real-time RT-PCR. Nat Protoc.

[48] Gene therapy clinical trials worldwide to 2017: An update

[49] Li SD, Huang L (2007). Non-viral Is Superior to Viral Gene Delivery. J Control Release.

[50] Zhu N, Liggitt D, Liu Y, Debs R (1993). Systemic gene expression after intravenous DNA delivery into adult mice. Science.

[51] Thierry AR, Lunardi-Iskandar Y, Bryant JL, Rabinovich P, Gallo RC, Mahan LC (1995). Systemic gene therapy: biodistribution and long-term expression of a transgene in mice. PNAS.

[52] Liu Y, Liggitt D, Zhong W, Tu G, Gaensler K, Debs R (1995). Cationic liposome-mediated intravenous gene delivery. J Biol Chem.

[53] Parker SE, Ducharme S, Norman J, Wheeler CJ (1997). Tissue distribution of the cytofectin component of a plasmid-DNA/cationic lipid complex following intravenous administration in mice. Hum Gene Ther.

[54] Liu F, Qi H, Huang L, Liu D (1997). Factors controlling the efficiency of cationic lipid-mediated transfection in vivo via intravenous administration. Gene Ther.

[55] Brigham KL, Meyrick B, Christman B, Magnuson M, King G, Berry LC (1989). In vivo transfection of murine lungs with a functioning prokaryotic gene using a liposome vehicle. Am J Med Sci.

[56] Mahato RI, Anwer K, Tagliaferri F, Meaney C, Leonard P, Wadhwa MS, Logan M, French M, Rolland A (1998). Biodistribution and gene expression of lipid/plasmid complexes after systemic administration. Hum Gene Ther.

[57] McClarrinon M, Gilkey L, Watral V, Fox B, Bullock C, Fradkin L, Liggitt D, Roche L, Bussey LB, Fox E, Gorman C (1999). In Vivo Studies of Gene Expression via Transient Transgenesis Using lipid-DNA Delivery. DNA Cell Biol.

[58] Hofland HE, Nagy D, Liu JJ, Spratt K, Lee YL, Danos O, Sullivan SM (1997). In vivo gene transfer by intravenous administration of stable cationic lipid/DNA complex. Pharm Res.

[59] Song YK, Liu F, Chu S, Liu D (1997). Characterization of cationic liposome mediated gene transfer in vivo by intravenous administration. Hum Gene Ther.

[60] Barron LG, Gagné L, Szoka FC (1999). Lipoplex-mediated gene delivery to the lung occurs within 60 minutes of intravenous administration. Hum Gene Ther.

[61] Opanasopit P, Nishikawa M, Hashida M (2002). Factors affecting drug and gene delivery: effects of interaction with blood components. Crit Rev Ther Drug Carrier Syst.

[62] Badea I, Verrall R, Baca-Estrada M, Tikoo S, Rosenberg A, Kumar P, Foldvari M (2005). In vivo cutaneous interferon-gamma gene delivery using novel dicationic (gemini) surfactant-plasmid complexes. J Gene Med.

[63] Alqawlaq S, Sivak JM, Huzil JT, Ivanova MV, Flanagan JG, Beazely MA, Foldvari M (2014). Preclinical development and ocular biodistribution of gemini-DNA nanoparticles after intravitreal and topical administration: towards non-invasive glaucoma gene therapy. Nanomedicine.

[64] Chien PY, Wang Y, Carbonaro D, Lei S, Miller B, Sheikh S, Ali SM, Ahmad MU, Ahmad I (2005). Novel cationic cardiolipin analogue-based liposome for efficient DNA and small interfering RNA delivery in vitro and in vivo. Cancer Gene Ther.

[65] Wasungu L, Scarzello M, van Dam G, Molema G, Wagenaar A, Engberts JBFN, Hoekstra D (2006). Transfection mediated by pH-sensitive sugar-based gemini surfactants; potential for in vivo gene therapy applications. J Mol Med.

[66] Varanita T, Soriano ME, Romanello V, Zaglia T, Quintana-Cabrera R, Semenzato M, Menabò R, Costa V, Civiletto G, Pesce P, Viscomi C, Zeviani M, Di Lisa F, Mongillo M, Sandri M, Scorrano L (2015). The OPA1-dependent mitochondrial cristae remodeling pathway controls atrophic, apoptotic, and ischemic tissue damage. Cell Metab.

[67] Del Dotto V, Fogazza M, Lenaers G, Rugolo M, Carelli V, Zanna C (2018). OPA1: how much do we know to approach therapy?. Pharmacol Res.

[68] Dotto V, Fogazza M, Carelli V (2018). Eight human OPA1 isoforms, long and short: What are they for?. Bioenergetics.

[69] Petit L, Khanna H, Punzo C (2016). Advances in gene therapy for diseases of the eye. Hum Gene Ther.

[70] Yu-Wai-Man P, Votruba M, Burté F, La Morgia C, Barboni P, Carelli V (2016). A neurodegenerative perspective on mitochondrial optic neuropathies. Acta Neuropathol (Berl).

[71] Foldvari M, Chen DW, Nafissi N, Calderon D, Narsineni L, Rafiee A (2016). Non-viral gene therapy: gains and challenges of non-invasive administration methods. J Control Release.

[72] Pal A, Datta S, Aswal VK, Bhattacharya S (2012). Small-angle neutron-scattering studies of mixed micellar structures made of dimeric surfactants having imidazolium and ammonium headgroups. J Phys Chem B.

[73] Rodriguez-Pulido A, Aicart E, Llorca O, Junquera E (2008). J Phys Chem B.

[74] Delgado AV (2002). Interfacial electrokinetics and electrophoresis.

[75] Oshima H, Furusawa K (1998). Electrical phenomena at interfaces fundamentals: measurements and applications.

