# Supplementary material for: Transgene expression in mice of the Opa1 mitochondrial transmembrane protein through bicontinuous cubic lipoplexes containing gemini imidazolium surfactants
Source: J Nanobiotechnology. 2021 Dec 18;19:425. doi: 10.1186/s12951-021-01167-x (PMC8684174; doi:10.1186/s12951-021-01167-x)
Supplement: Supplementary file 1 — Additional file 1. Supporting material. [file 12951_2021_1167_MOESM1_ESM.pdf]

SUPPORTING MATERIAL

Transgene expression in mice of the Opa1 mitochondrial transmembrane protein through bicontinuous cubic lipoplexes containing gemini imidazolium surfactants

Mónica Muñoz-Úbeda[1,2,*] Martina Semenzato[3], Anais Franco-Romero[3], Elena Junquera[2], Emilio Aicart[2], Luca Scorrano[3] and Iván López-Montero[1,2,*].

1 Instituto de Investigación Biomédica Hospital 12 de Octubre (imas12), Madrid, Spain

2 Dpto. Química Física, Universidad Complutense de Madrid, Spain.

3 Fondazione per la Ricerca Biomèdica Avanzata, Venetian Institute of Molecular Medicine (VIMM), Padova, Italy.

Corresponding author: monicamubeda@quim.ucm.es, ivanlopez@quim.ucm.es

Determination of the effective charge of the gemini cationic lipid and the MSCV-OPA1 plasmid. The composition of the mixed lipid acting as cationic gene vector is given in terms of its molar fraction (α) of the cationic lipid in the total lipid, while in the lipoplex, the composition may be expressed by: a) the total lipid to DNA mass ratio, defined as ($m_L/m_D = (m_L^+ + m_L^0)/m_D$), where m_L, m_L^+, m_L^0 and m_D are the masses of the total mixed lipid, $(C_{16}Im)_2(C_4O)$, DOPE, DSPE-PEG and DNA, respectively, or b) the effective charge ratio (ρ_{eff}) expressed as the ratio between the charges of positive $(C_{16}Im)_2(C_4O)$ mixed lipid and negative DNA phosphate groups. All these quantities are related by the following two equations:

$$\alpha = \frac{m_{L^+} / M_{L^+}}{(m_{L^+} / M_{L^+}) + (m_{L^0} / M_{L^0})} \quad (1)$$

$$\rho_{eff} = \frac{n^+}{n^-} = \frac{q^+_{eff,L^+}(m_{L^+} / M_{L^+})}{q^-_{eff,D}(m_D / M_D)} \quad (2)$$

where n^+ and n^- are the number of moles of positive and negative charges of $(C_{16}Im)_2(C_4O)$ and DNA respectively; (q^+_{eff,L^+}) and ($q^-_{eff,D}$) are the effective charges of $(C_{16}Im)_2(C_4O)$ and plasmid DNA (pDNA, MSCV-OPA1 in our case) per bp; and M_L^0, M_L^+ and M_D are the molecular weight of the DOPE, $(C_{16}Im)_2(C_4O)$ and MSCV-OPA1 per bp, respectively.

The eletroneutrality ratio of the lipoplex $((m_L^+ + m_L^0) / m_D)_\Phi$ is reached for a particular formulation at which the positive charges of the mixed lipid and those negative of DNA balance ($\rho_{eff} = 1$). Values of ρ_{eff} higher than the electroneutrality ratio are required for lipoplexes to become a potentially cell transfecting agent as the positively charged lipoplexes allow them crossing the negatively charged cell membranes.[1] The electroneutrality ratio can be accurately determined by measuring the zeta potential (ζ) of lipoplexes as a function of (m_L/m_D) (see **Figure 1A**). A sign inversion on the charge in the (ζ) sigmoidal plots occurs at $(m_L/m_D)_\Phi$ and this value is related to α through the equations (1-2) by:

$$\left(\frac{m_L}{m_D}\right)_\Phi = \left(\frac{m_{L^+} + m_{L^0}}{m_D}\right)_\phi = \frac{q_D^-[\alpha M_{L^+} + (1-\alpha) M_{L^0}]}{q_L^+ \alpha M_D}$$

(3)

In general, linear DNAs, such as calf thymus DNA (ctDNA), have its negative charge totally available for the cationic lipid, i.e., $q_{linear\ D}$ = -2 per base pair. However, plasmid DNA remains in a supercoiled conformation[2-7] rendering a much less negative charge than its nominal one ($q^-_{eff,D} \ll -2/bp$). The determination of the effective charge of both, the cationic lipid (q^+_{eff,L^+})and the pDNA ($q^-_{eff,D}$) is required then to quantitatively formulate lipoplexes with different effective charge ratios (ρ_{eff}). For that, the effective charge of the $(C_{16}Im)_2(C_4O)$ (q^+_{eff,L^+}) was first determined using equation (3) and the experimental value of $(m_L/m_D)_\Phi$ measured from zeta potential (**Figure S2**), and assuming $q_{linear\ D}$ = -2/bp. The effective charge of MSCV-OPA1 ($q^-_{eff,D}$) is then obtained with equation (4) using the effective charge of the $(C_{16}Im)_2(C_4O)$ (q^+_{eff,L^+}) and the experimental electroneutrality value $(m_L/m_D)_\Phi$ for the $(C_{16}Im)_2(C_4O)$/DOPE/DSPE-PEG lipoplex containing MSCV-OPA1 plasmid DNA (**Figure 1**):

$$q^{-}_{eff,D} = \left(\frac{m_L}{m_D}\right)_{\Phi} \left(\frac{q^{+}_{L^+}\alpha M_D}{\alpha M_{L^+} + (1-\alpha) M_{L^0}}\right) \tag{4}$$

The effective charge ratio (ρ_{eff}) of the lipoplex at α = 0.2 is obtained by substituting (q^{+}_{eff,L^+}) and the MSCV-OPA1 ($q^{-}_{eff,D}$) in equation (2).

REFERENCES

(1) Dias, R. S.; Lindman, B. DNA Interaction with Polymers and Surfactants; *Wiley & Sons: Hoboken, NJ* **2008**.

(2) Muñoz-Úbeda, M.; Misra, S. K.; Barrán-Berdón, A. L.; Aicart-Ramos, C.; Sierra, M. B.; Biswas, J.; Kondaiah, P.; Junquera, E.; Bhattacharya, S.; Aicart, E. Why is less cationic lipid required to prepare lipoplexes from plasmid DNA than linear DNA in gene therapy? *J. Am. Chem. Soc*. **2011**, *133*, 18014-18017.

(3) Barrán-Berdón, A. L.; Misra, S. K.; Datta, S.; Muñoz-Úbeda, M.; Kondaiah, P.; Junquera, E.; Bhattacharya, S.; Aicart, E. Cationic gemini lipids containing polyoxyethylene spacers as improved transfecting agents of plasmid DNA in cancer cells. *J. Mater. Chem. B* **2014**, *2*, 4640-4652.

(4) Martinez-Negro, M.; Guerrero-Martinez, A.; Garcia-Rio, L.; Domenech, O.; Aicart, E.; de Ilarduya, C. T.; Junquera, E. Multidisciplinary approach to the transfection of plasmid DNA by a nonviral nanocarrier based on a gemini-bolaamphiphilic hybrid lipid. *ACS Omega* **2018**, *3*, 208-217.

(5) Misra, S. K.; Muñoz-Úbeda, M.; Datta, S.; Barrán-Berdón, A. L.; Aicart-Ramos, C.; Castro-Hartmann, P.; Kondaiah, P.; Junquera, E.; Bhattacharya, S.; Aicart, E. Effects of a delocalizable cation on the headgroup of gemini lipids on the lipoplex-type nano-aggregates directly formed from plasmid DNA. *Biomacromolecules* **2013**, *14*, 3951-3963.

(6) Lyubchenko, Y. L.; Shlyakhtenko, L. S. Visualization of supercoiled DNA with atomic force microscopy in situ. *Proc. Natl. Acad. Sci. USA* **1997**, *94*, 496-501.

(7) Foldvari, M.; Badea, I.; Wettig, S.; Verrall, R.; Bagonluri, M. Structural characterization of novel gemini non-viral DNA delivery systems for cutaneous gene therapy. *J. Exp. Nanosci*. **2006**, *1*, 165-176.

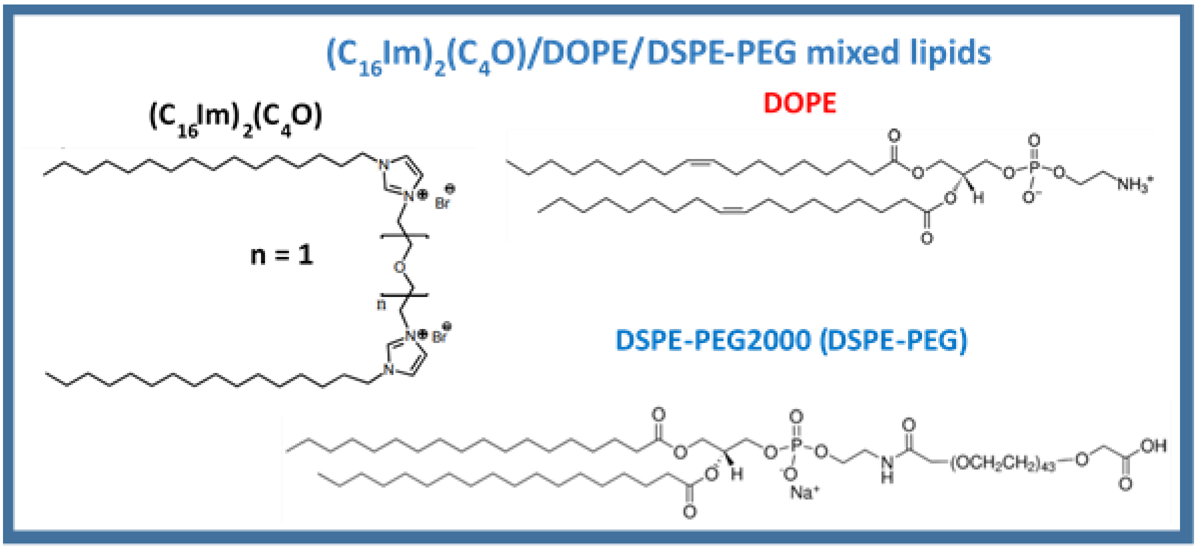

Scheme S1: Structures of $(C_{16}Im)_2(C_4O)$ gemini cationic lipid, the zwitterionic phospholipid DOPE and the polyethylene-glycol DSPE-PEG2000 (DSPE-PEG).

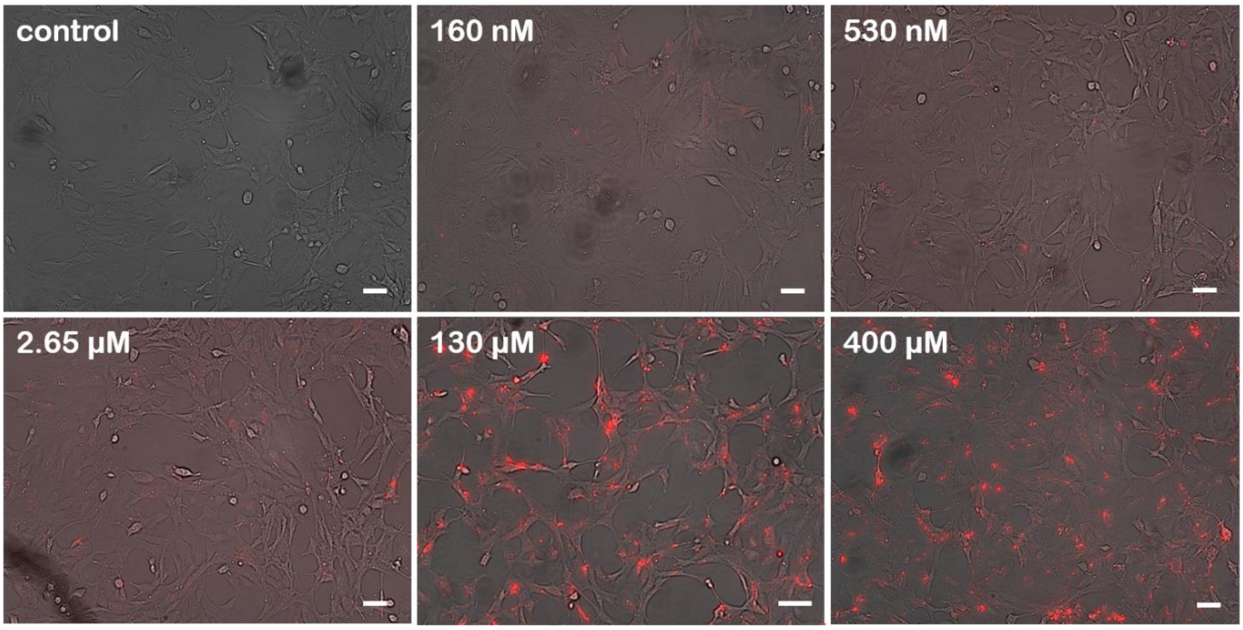

Figure S1. Uptake of $(C_{16}Im)_2(C_4O)$/DOPE/DSPE-PEG/OPA1 lipoplexes labeled with the fluorescent dye DiR' (red channel) into MEFs wt after 24 h incubation and at different concentration. Scale bars are 20 µm.

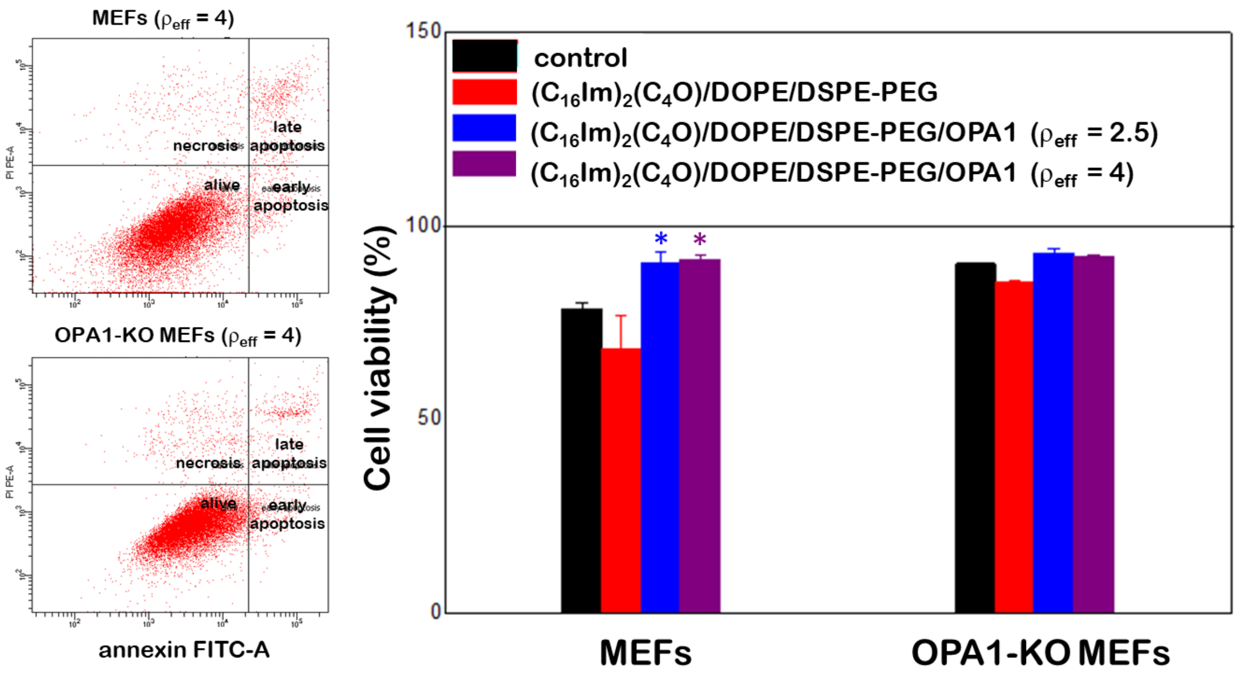

Figure S2. Apoptosis after 24h of treatment was measured by flow cytometry through Annexin V-FITC and PI staining. Representative plots for MEFs wt ($\rho_{eff} = 4$) and OPA1-KO MEFs ($\rho_{eff} = 4$) are shown in the left panel. The percentage of alive cells is indicated in the right panel. The data are presented as the means ± the SD from three separate experiments (* $p < 0.05$).

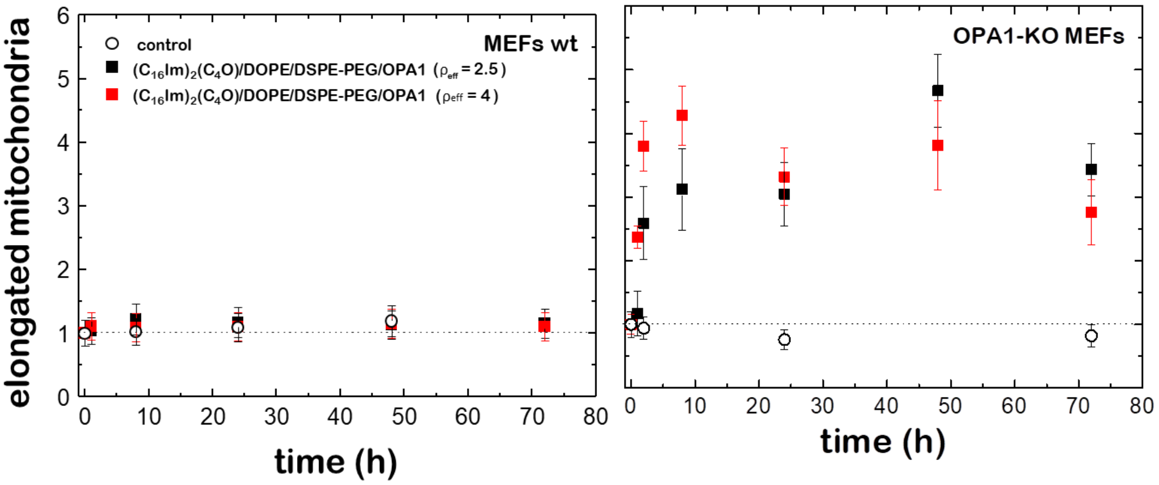

Figure S3. Time evolution of the elongated mitochondria ratio with respect to initial elongated mitochondria for MEFs wt (left panel) and OPA1-KO MEFs (right panel) after transfection with $(C_{16}Im)_2(C_4O)$/DOPE/DSPE-PEG/OPA1 lipoplexes at ρ_{eff} = 2.5 (black squares) and 4 (red squares). As a control, the mitochondrial phenotype was also imaged in the absence of treatment (hollow symbols). See Methods section for details. Student's t-test was performed to measure the significance of statistical difference between the different groups and the negative control (in the absence of treatment). $p < 0.05$ was considered statistically significant.

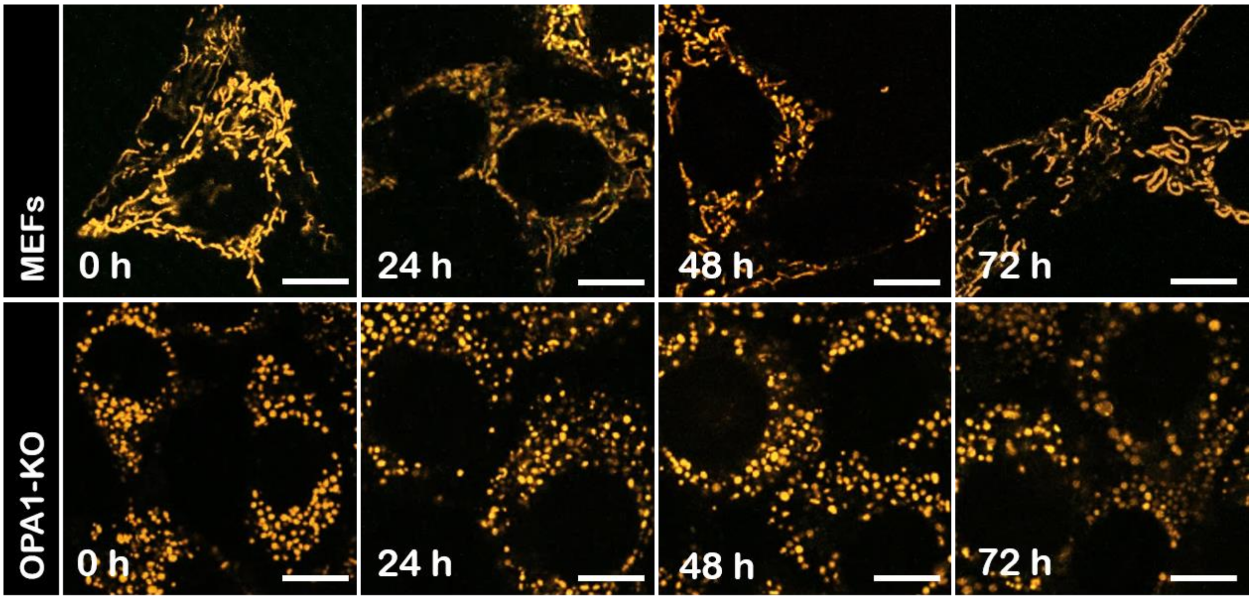

FIGURE S4. Confocal fluorescence microscopy images MEFs wt (upper row) and OPA1-KO MEFs (lower row) at different time intervals in the absence of treatment. The mitochondrial network was labeled with TMRM (1 µM). Scale bars are 10 µm.

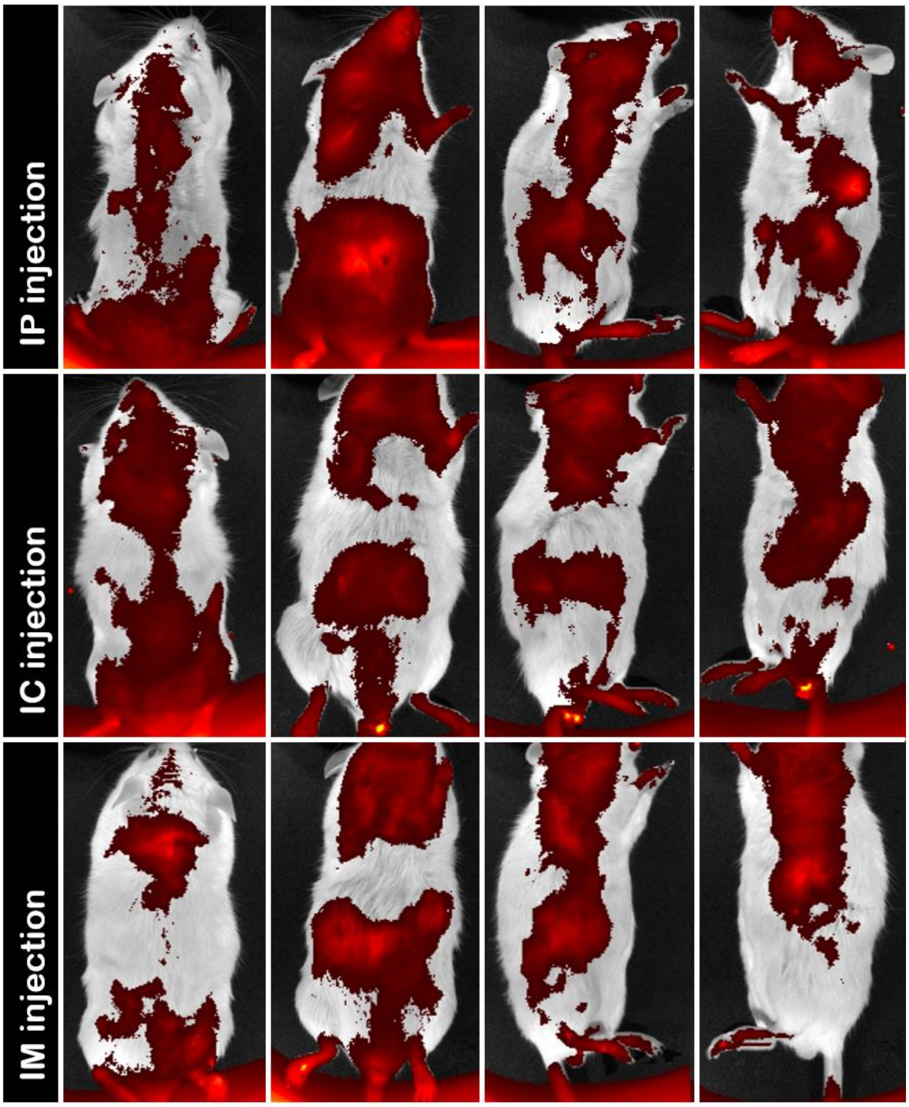

Figure S5. *In vivo* biodistribution of DiR'-labeled $(C_{16}Im)_2(C_4O)$/DOPE/DSPE-PEG/OPA1 lipoplexes in CD-1 mice after 4 weeks with treatment via intraperitoneal (IP), intracardiac (IC) and intramuscular (IM) administration.

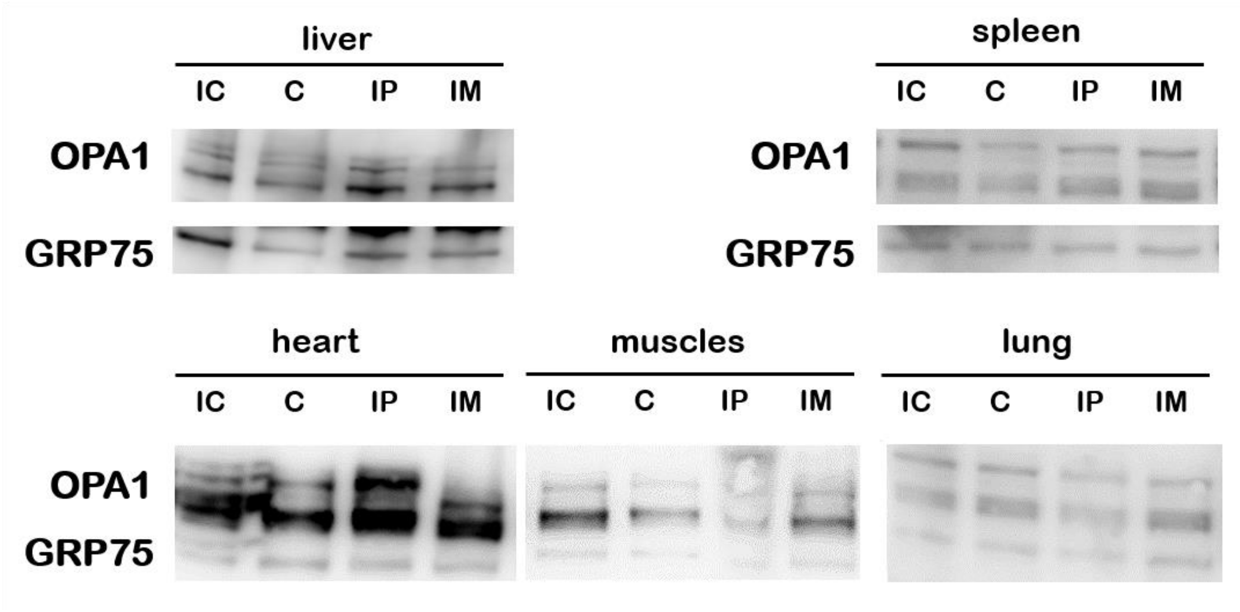

Figure S6. Western blot of Opa1 and GRP75 protein levels in liver, spleen, heart, muscles and lung from CD-1 mice after 48 h of transfection with $(C_{16}Im)_2(C_4O)$/DOPE/DSPE-PEG/OPA1 lipoplexes (ρ_{eff} = 4). Different lanes correspond to the groups without treatment (C) and with treatment via intraperitoneal (IP), intracardiac (IC) and intramuscular (IM) administration.
